# DHA alleviates diet-induced skeletal muscle fiber remodeling via FTO/m^6^A/DDIT4/PGC1α signaling

**DOI:** 10.1186/s12915-022-01239-w

**Published:** 2022-02-08

**Authors:** Wei Chen, Yushi Chen, Ruifan Wu, Guanqun Guo, Youhua Liu, Botao Zeng, Xing Liao, Yizhen Wang, Xinxia Wang

**Affiliations:** 1 College of Animal Sciences, Zhejiang University, No. 866 Yuhangtang Road, Hangzhou, 310058 Zhejiang province China; 2 Key Laboratory of Molecular Animal Nutrition (Zhejiang University), Ministry of Education, Hangzhou, 310058 China; 3 Key Laboratory of Animal Nutrition and Feed Science (Eastern of China), Ministry of Agriculture and Rural Affairs, Hangzhou, 310058 China; 4 Key Laboratory of Animal Feed and Nutrition of Zhejiang Province, Hangzhou, 310058 China

**Keywords:** DHA, FTO, Muscle fiber, Obesity, PGC1α

## Abstract

**Background:**

Obesity leads to a decline in the exercise capacity of skeletal muscle, thereby reducing mobility and promoting obesity-associated health risks. Dietary intervention has been shown to be an important measure to regulate skeletal muscle function, and previous studies have demonstrated the beneficial effects of docosahexaenoic acid (DHA; 22:6 ω-3) on skeletal muscle function. At the molecular level, DHA and its metabolites were shown to be extensively involved in regulating epigenetic modifications, including DNA methylation, histone modifications, and small non-coding microRNAs. However, whether and how epigenetic modification of mRNA such as N6-methyladenosine (m6A) mediates DHA regulation of skeletal muscle function remains unknown. Here, we analyze the regulatory effect of DHA on skeletal muscle function and explore the involvement of m^6^A mRNA modifications in mediating such regulation.

**Results:**

DHA supplement prevented HFD-induced decline in exercise capacity and conversion of muscle fiber types from slow to fast in mice. DHA-treated myoblasts display increased mitochondrial biogenesis, while slow muscle fiber formation was promoted through DHA-induced expression of PGC1α. Further analysis of the associated molecular mechanism revealed that DHA enhanced expression of the fat mass and obesity-associated gene (FTO), leading to reduced m6A levels of DNA damage-induced transcript 4 (*Ddit4*). *Ddit4* mRNA with lower m6A marks could not be recognized and bound by the cytoplasmic m6A reader YTH domain family 2 (YTHDF2), thereby blocking the decay of *Ddit4* mRNA. Accumulated *Ddit4* mRNA levels accelerated its protein translation, and the consequential increased DDIT4 protein abundance promoted the expression of PGC1α, which finally elevated mitochondria biogenesis and slow muscle fiber formation.

**Conclusions:**

DHA promotes mitochondrial biogenesis and skeletal muscle fiber remodeling via FTO/m^6^A/DDIT4/PGC1α signaling, protecting against obesity-induced decline in skeletal muscle function.

**Supplementary Information:**

The online version contains supplementary material available at 10.1186/s12915-022-01239-w.

## Background

The incidence of obesity has been rising steadily over the past few decades, mainly due to excessive energy intake. By 2025, the global obesity rate will be 18% for men and more than 21% for women [1]. As a result, obesity and its related metabolic diseases are increasingly becoming a chronic disease with a health threat and economic burden. Obesity can impair skeletal muscle function, such as muscular atrophy, insulin resistance, and a shift from slow to fast muscle fiber types, thereby reducing mobility and further increasing the health risks associated with obesity [2, 3]. Skeletal muscle comprises different muscle fiber types, which can be divided into slow-twitch and fast-twitch fibers. The proportion of the different fiber types determines contractile and metabolic properties of muscle tissue [4]. The transition of muscle fiber type from slow-twitch to fast-twitch caused by obesity leads to the change of metabolism mode and the decrease of insulin sensitivity in skeletal muscle, which exacerbates the systemic metabolic imbalance in turn [5]. Recent studies have reported that exercise and nutrition regulation can reverse the transition of skeletal muscle fiber type caused by obesity and restore its exercise and metabolic function, so as to improve systemic metabolism [6, 7]. Therefore, it is meaningful to improve the function of skeletal muscle in obesity by nutritional pathway.

Recently, interest in the biological functions of Omega-3 (ω-3) polyunsaturated fatty acids (PUFAs) has escalated because of their various roles in health promotion and disease risk reduction [8]. DHA is an essential fatty acid that is ubiquitous in marine animals and plant plankton [9]. Previous studies have reported that the bioactivities of DHA, such as improvement in brain function [10], antitumor activity [11], regulation of lipid metabolism [12], regulation of glucose metabolism [13], anti-inflammatory effect [14], and improvement on exercise training and performance [15]. Increasing evidence also supports the beneficial effects of DHA on skeletal muscle function, such as alleviating muscular atrophy [16], ameliorating endurance exercise capacity [17], and contributing in recovery from exhaustion [18]. However, there are some contradictions in the current reports on the effects of DHA on skeletal muscle under different physiological conditions, and its molecular mechanism needs to be further explored.

Studies on the molecular mechanisms have shown that DHA and its metabolites are extensively involved in regulating epigenetic modifications, including DNA methylation, histone modifications, and small non-coding microRNAs [19]. However, how epigenetic modification mediates the regulation of DHA on skeletal muscle remains unclear. N^6^-methyladenosine (m^6^A), the most abundant mRNA modification in eukaryotes, has recently emerged as a significant post-transcriptional regulator. m^6^A methylation plays a crucial role in mediating many important biological processes such as development, metabolism, and disease [20]. Considering that several studies have found that m^6^A regulates skeletal muscle function, such as myogenesis [21, 22], lipid deposition [23], and muscle regeneration [24], we wanted to explore whether DHA could influence m^6^A level in skeletal muscle and improve its function through RNA modification.

## Results

### DHA alleviates diet-induced obesity and metabolic dysfunctions

**Fig. 1:**
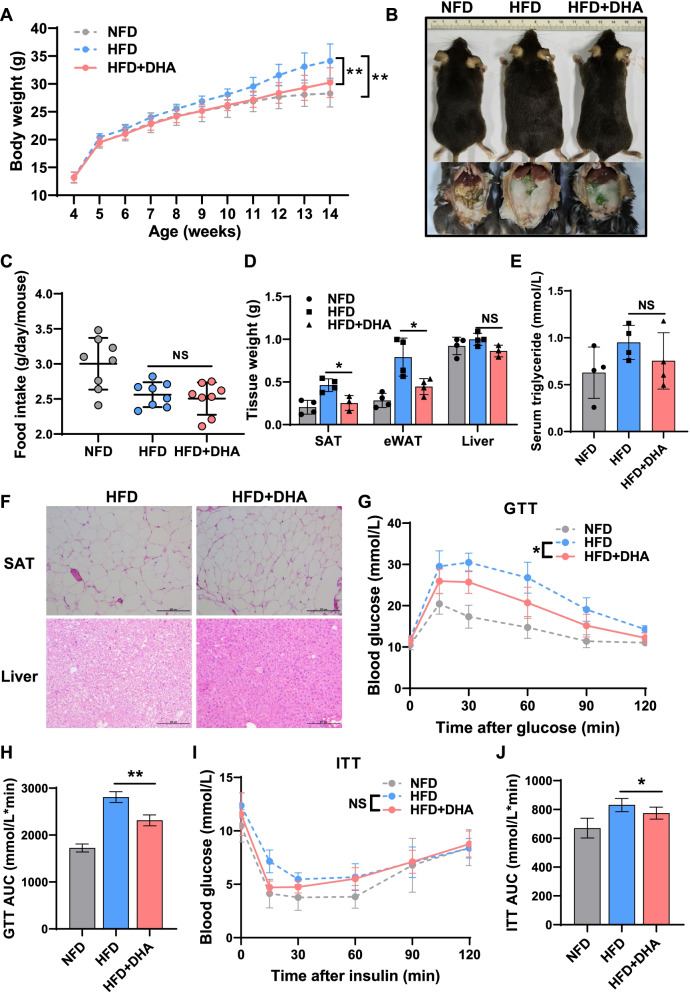
The dietary supplement of DHA prevents HFD-induced obesity. **A** Changes in body weight across time (*n* = 8). **B** Representative pictures of mice in three groups. **C** The food intake in three groups (*n* = 8). **D** Weight of subcutaneous adipose tissue (SAT) and epididymal white adipose tissue (eWAT) from mice in three groups, NS, not statistically significant (*n* = 3 or 4). **E** Basic serum triglyceride level of mice in three groups (*n* = 4). **F** Representative pictures of SAT and liver tissue from mice fed HFD and HFD+DHA. Scale bar: 200 μm. **G** Intraperitoneal glucose tolerance test (GTT) (*n* = 6), significant differences are shown between HFD and HFD+DHA (∗). **H** Area under curve (AUC) of GTT. **I** insulin tolerance test (ITT) (*n* = 6), significant differences are shown between HFD and HFD+DHA (∗∗). **J** AUC of ITT (*n* = 6). Statistical analysis was performed using one-way ANOVA with time-repeated measurements (Fig. 1A, G, and I), two-tailed paired Student’s *t* tests (Fig. 1C–E, H and J)

To assess the effect of DHA on skeletal muscle function in a diet-induced obesity (DIO), we fed the mice with normal fat diet (NFD), high-fat diet (HFD), or high-fat diet supplemented with DHA (HFD+DHA) for 10 weeks. We found that the final body weight of mice in HFD group was significantly higher than those fed NFD, and this showed that our obesity model is successfully constructed (Fig. 1A). Mice fed with HFD+DHA gained less body weight and fat accumulation than those in HFD group (Fig. 1A, B), while the food intake showed no significant difference (Fig. 1C). Consistent with the weight results, the subcutaneous adipose tissue (SAT) and epididymal white adipose tissue (eWAT) of HFD+DHA group were smaller and weighed less than those of HFD group, while there was no difference in liver weight (Fig. 1D). However, there was no significant difference in serum triglyceride content between HFD group and HFD+DHA group (Fig. 1E). Histological analysis showed that mice had markedly reduced adipocyte size and liver lipid content in HFD+DHA group compared with those in HFD group (Fig. 1F). To investigate the effect of three dietary regimens on metabolism in mice, we next tested the glucose tolerance test (GTT) and insulin tolerance test (ITT) in three groups. After glucose injection, the blood glucose declined markedly faster in HFD+DHA group when compared with those in HFD group (Fig. 1G). The results were also verified by the area under curve (AUC) value of GTT (Fig. 1H). Similar results were also shown in ITT data. Although DHA supplementation had no significant effect on ITT data, AUC results showed that DHA increased insulin sensitivity in obese mice (Fig. 1I, J). These results above indicate that DHA protects mice against HFD-induced obesity and the related metabolic dysfunctions partly.

### DHA improves skeletal muscle function under the condition of HFD

**Fig. 2:**
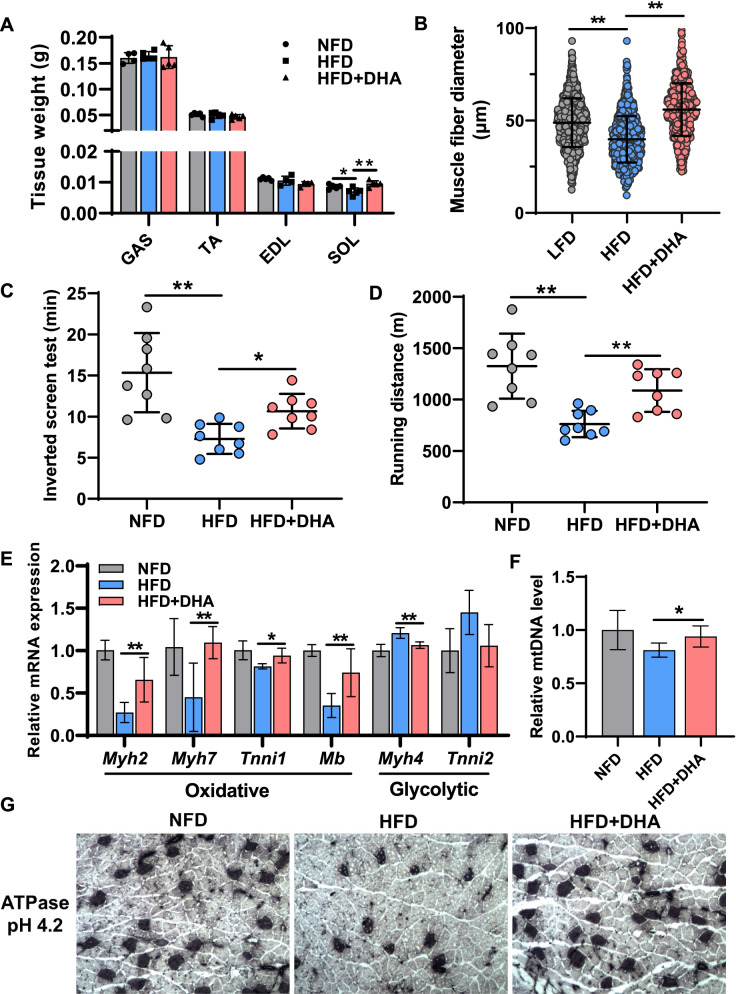
DHA improves skeletal muscle function under the condition of HFD. **A** Weight of gastrocnemius (GAS), tibialis anterior (TA), extensor digitorum longus (EDL), and soleus (SOL) from mice in three groups (*n* = 4 or 5). **B** Diameter distribution of muscle fibers in TA from mice in three groups (μm) (*n* > 500). **C** Inverted screen test of mice in three groups (*n* = 8). **D** Running distance test of mice in three groups (*n* = 8). **E** Relative mRNA expression of oxidative and glycolytic muscle fiber type markers measured by qPCR in GAS from mice in three groups (*n* = 6). **F** Relative mitochondrial content of mice in three groups (*n* = 6). **G** Metachromatic ATPase staining of GAS to detect type I muscle fibers (black). Statistical analysis was performed using two-tailed paired Student’s *t* tests

Next, we investigated the effects of DHA on the function of skeletal muscle. No significant differences were observed in morphology of various types of muscle in three groups (Additional File 1: Fig. S1A). The HFD alone resulted in weight loss in the mice soleus (SOL), while additional DHA supplementation under HFD condition alleviated this muscle loss (Fig. 2A). By quantifying the hematoxylin-eosin staining (H&E) sections of the gastrocnemius (GAS) in mice (Additional File 1: Fig. S1B), we found that HFD caused muscle fiber atrophy in mice, while DHA reversed this phenomenon (Fig. 2B). To further investigate the effects of DHA on skeletal muscle contraction properties, we then tested the exercise capacity of mice. We found that DHA increased the time of inverted screen test and the running distance (Fig. 2C, D), which indicated DHA could improve endurance exercise performance. It is well-known that skeletal muscle is composed of various types of muscle fibers, among which, slow muscle fibers contain more mitochondria, and their metabolic pattern is dominated by oxidative phosphorylation. Therefore, the higher ratio of slow muscle fibers, the stronger the exercise endurance. To ascertain whether the altered endurance exercise performance was associated with changes in muscle fiber types, we tested the muscle fiber types and gene expression associated with metabolic patterns. We found that DHA upregulated slow-twitch fiber-associated genes (*Myh2*, *Myh7*, *Tnni1*, and *Mb*), whereas it downregulated fast-twitch fiber-associated genes, including *Myh4* and *Tnni2* (Fig. 2E). In addition, DHA also led to more mitochondria content in the skeletal muscle of mice as indicated by mitochondrial DNA (mtDNA) level (Fig. 2F). In agreement with gene expression and mtDNA level, ATPase staining showed that HFD caused a markedly decline of slow-twitch muscle fiber proportion in GAS, while adding DHA under HFD condition increased the proportion of slow-twitch muscle fiber (Fig. 2G). The above results suggest that DHA improves skeletal muscle function under HFD, which may be due to the transition of glycolytic to oxidative muscle fibers induced.

### DHA supplementation changes the lipid composition in muscle tissue

**Fig. 3:**
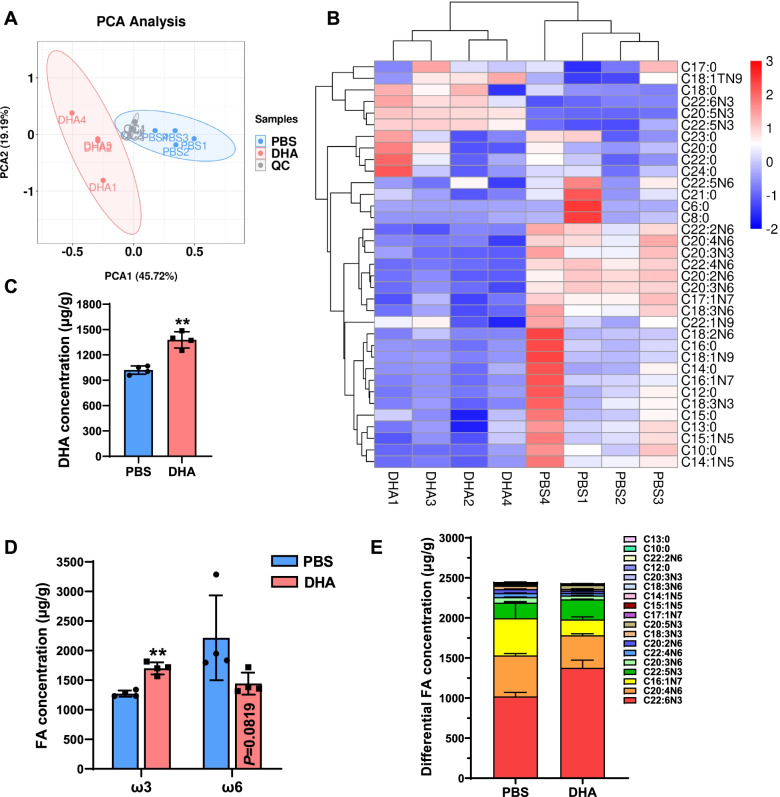
Administration of DHA changed the lipid composition of muscle. **A** PCA of lipodomics from mice GAS in PBS and DHA group. **B** Heatmaps showing normalized concentration of medium- and long-chain fatty acids in GAS of PBS and DHA group (*n* = 4). **C** DHA concentration in GAS (fatty acid content per gram of GAS) (*n* = 4). **D** Concentration of ω3 and ω6 fatty acids (FA) in GAS (*n* = 4). **E** The composition of fatty acids with altered concentration (*P* < 0.05) (*n* = 4). Statistical analysis was performed using two-tailed paired Student’s *t* tests

In order to further explore the mechanism of DHA supplementation affecting skeletal muscle function, we first tested whether DHA supplementation changed the fatty acid content of muscle tissue in the early stage of gavage. Specifically, through targeted metabonomics, we analyzed the changes of medium and long-chain fatty acids in mice gastrocnemius muscle. Principal component analysis (PCA) showed that mice fed with DHA showed different composition of fatty acids in muscle from the control group (Fig. 3A). Among these fatty acids, the concentrations of 18 fatty acids changed (3 upregulated and 15 downregulated) (Additional file 2), including the increased DHA (C22:6N3) (Fig. 3B, C). So the administrated DHA was indeed digested and retained in the muscle tissues. In addition, DHA supplementation significantly increased the overall ω3 fatty acid concentration (*P*<0.01) and decreased the ω6 fatty acid concentration that nearly reached statistical significance (*P* = 0.08 for trend) (Fig. 3D). Although the concentrations of various fatty acids have changed, DHA accounts for the largest proportion (Fig. 3E). Therefore, we speculated that DHA may play a major role in regulating muscle function and further verified that in cell model.

### DHA increases aerobic oxidation and mitochondrial biogenesis in C2C12

**Fig. 4:**
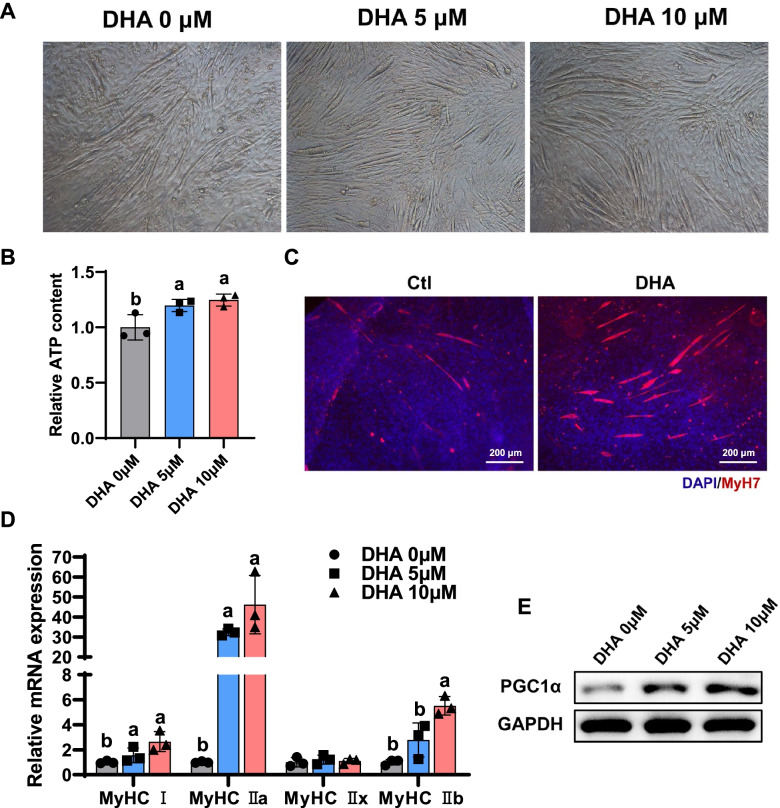
DHA increases aerobic oxidation and mitochondrial biogenesis in C2C12. **A** Myotube formation of C2C12 myoblasts with DHA treatment (0, 5, 10 μM, 4 days). **B** ATP levels from early stage of myogenic differentiation (*n* = 3, 48 h). **C** Representative images of slow muscle fiber (MyHC7) immunofluorescent staining in C2C12 myoblasts after 4 day differentiation. Scale bar: 200 μm. **D** qPCR measured different types of muscle fiber gene expression (*n* = 3, 48 h). **E** Western blot analysis of PGC1α expression from C2C12 treated with BSA or DHA (5, 10 μM). Different letters indicate significant differences between different doses of DHA treatment for 48 h (*P* < 0.05). Statistical analysis was performed using one-way ANOVA

To explore the intracellular mechanism for DHA-induced fiber-type transition, we conducted studies in C2C12 myoblasts with various concentrations of DHA. Cell proliferation assay showed that 100 μM or more of DHA was cytotoxic to C2C12 cells (Additional File 1: Fig. S2A). To test the effect of critical concentrations of DHA on cells, higher concentrations of DHA (50, 100 μM) were added to C2C12 myoblasts under differentiated medium supplemented with 2% horse serum. Bright field and H&E staining showed that DHA with 50 or 100 μM inhibited the formation of myotubules and downregulated myosin heavy chain (MyHC) expression. At the early stage of myogenesis (2 days after differentiation), DHA treatment also inhibited the expression of *Myod1* and *Myog* genes, which are important genes during myogenesis (Additional File 1: Fig. S2B-D). Due to its bidirectional differentiation potential, C2C12 can be induced into myocytes or adipocytes. Previous studies have shown that high concentrations of fatty acids increase the expression of lipid-generating genes [25], so we hypothesized that high concentrations of DHA promoted the differentiation of C2C12 into adipocytes. Consistent with our hypothesis, we found that high concentrations of DHA increased intracellular lipid droplets even in myoblast differentiation medium (Additional File 1: Fig. S2E, F). Meanwhile, DHA treatment resulted in increased expression of genes associated with adipogenic differentiation in C2C12 cells, indicating that high DHA concentrations indeed promoted the differentiation of C2C12 into adipocytes (Additional File 1: Fig. S2G). These results contradict studies that DHA improves skeletal muscle function in vivo, so we considered whether the higher concentrations of DHA in cell culture would not correspond to the physiological concentration of DHA in vivo. Other studies have shown that the concentration of DHA in and skeletal muscle is about 5 μM [26], so we treated C2C12 with low concentrations of DHA (5, 10 μM). We found that low concentrations of DHA had no effect on myoblast differentiation (Fig. 4A) or adipogenesis (Additional File 1: Fig. S2H, I), which was confirmed by the related gene expression (Additional File 1: Fig. S2J). Intriguingly, low concentrations of DHA significantly increased ATP content in myoblasts (Fig. 4B). DHA treatment increased the number of slow muscle fibers and the expression of oxidative and glycolytic muscle fiber type markers (Fig. 4C, D). Since peroxisome proliferator-activated receptor-γ coactivator 1α (PGC1α) can regulate mitochondria biogenesis and muscle fiber transformation [27], we want to know whether DHA regulated mitochondrial number and slow muscle fiber formation through PGC1α pathway. Western blotting assays confirmed that the expression of PGC1α was promoted after low concentrations of DHA treatment (Fig. 4E). Taken together, these results showed that high concentrations of DHA (50, 100 μM) inhibited myogenesis, while low concentrations of DHA (5, 10 μM) changed muscle fiber composition. Further study showed DHA-mediated muscle fiber transformation might be through the PGC1α pathway.

### DHA reduces m^6^A levels by increasing FTO protein expression

**Fig. 5:**
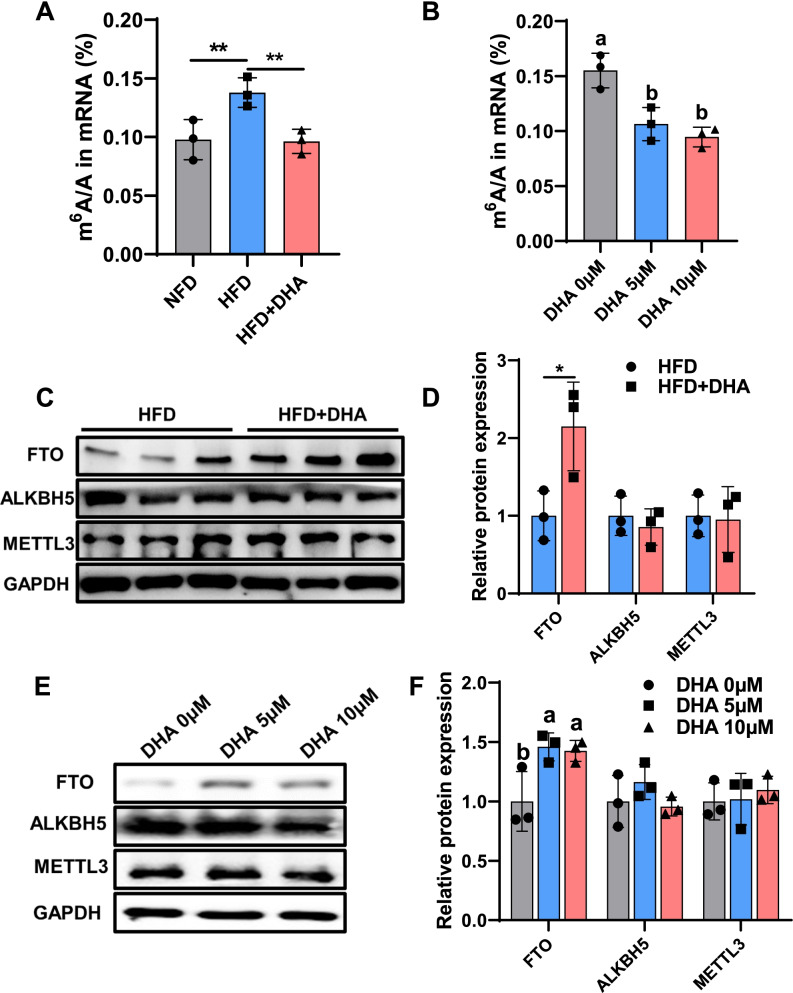
DHA reduces m6A levels by increasing FTO protein expression. **A** LC-MS/MS quantification of the m^6^A/A in mRNA of GAS from mice fed HFD or HFD+DHA (*n* = 3). **B** LC-MS/MS quantification of the m^6^A/A in mRNA of myoblasts with different concentration of DHA (*n* = 3). Western blot analysis and quantification of major m^6^A methyltransferase and demethylase expression in **C**, **D** skeletal muscle and **E, F** myoblasts (*n* = 3). Different letters indicate significant differences between different doses of DHA treatment for 48 h (*P* < 0.05). Statistical analysis was performed using one-way ANOVA (Fig. 5B, F) and two-tailed paired Student’s *t* tests (Fig. 5A, D)

Recent reports have demonstrated that m^6^A is widely involved in diverse eukaryotic biological processes, including cell differentiation [28, 29] and metabolic reprogramming [30, 31]. Nutrients can play their physiological functions by regulating m^6^A levels, such as epigallocatechin gallate [32], curcumin [33, 34], and NADP [35]. DHA previously has been shown to regulate DNA methylation [36], which raises the question of whether DHA function is also related to RNA methylation. We first measured total m^6^A modified mRNA levels using liquid chromatography-tandem mass spectrometry (LC-MS/MS) and found that DHA significantly reduced the total m^6^A levels in mRNA from both skeletal muscle tissues and myoblasts (Fig. 5A, B). m^6^A dot blot also showed consistent results (Additional File 1: Fig. S2K.). By detecting the expression of major m^6^A methyltransferase and demethylase in muscle tissues and myoblasts, we found that DHA downregulated m^6^A level mainly through promoting the protein expression of m^6^A demethylase FTO in vivo and in vitro (Fig. 5C–F).

### DHA increases PGC1α expression through FTO

**Fig. 6:**
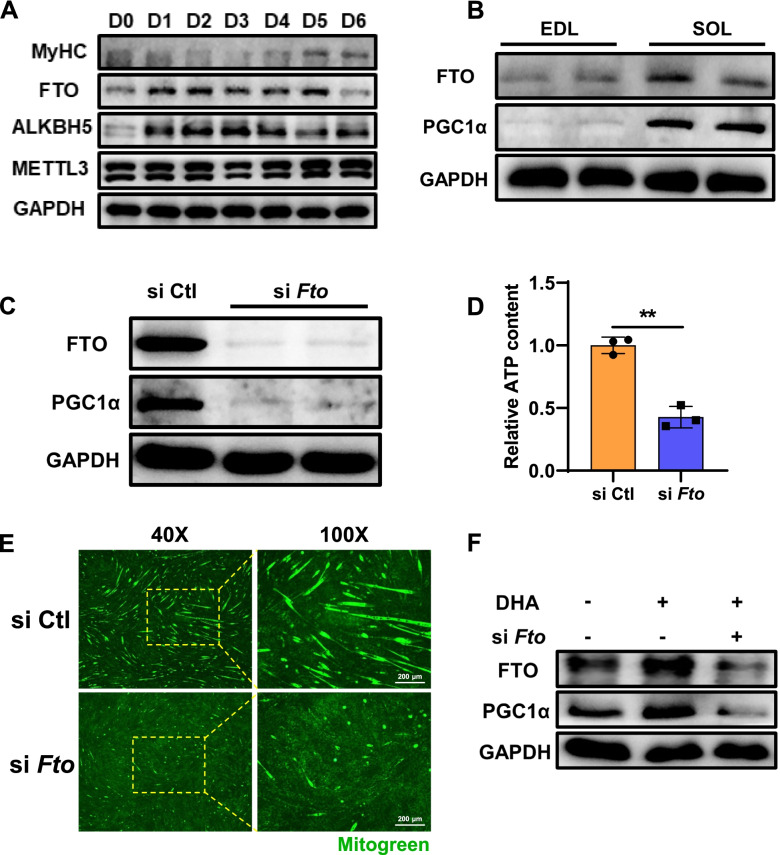
DHA increases PGC1α expression through FTO. **A** Western blot analysis of major m^6^A methyltransferase and demethylase expression profile during myogenesis (*n* = 3). **B** Western blot analysis of FTO and PGC1α in fast-twitch muscle fiber (EDL) and slow-twitch muscle fiber (SOL) (*n* = 3). **C** The protein expression of PGC1α, **D** relative ATP content, and **E** mitogreen staining was tested in C2C12/siCtl and C2C12/si*Fto* (*n* = 3). Scale bar: 200 μm. **F** Western blot analysis of FTO and PGC1α expression after DHA treatment and *Fto* silencing (*n* = 3). Statistical analysis was performed using two-tailed paired Student’s *t* tests

To determine the role of FTO in DHA-induced skeletal muscle fiber switching in vivo, we first examined the expression profile of FTO during myogenesis. The expression level of FTO was increased during myoblast differentiation (Fig. 6A). SOL is known as a typical slow-twitch muscle, whereas extensor digitorum longus (EDL) is a typical fast-twitch muscle, and FTO was highly expressed in slow muscle fibers in sync with PGC1α expression (Fig. 6B). The above results showed that FTO may play a key role in myogenesis and muscle fiber type remodeling. To further confirm this hypothesis, we first validated that silencing of FTO substantially reduced the protein expression of PGC1α (Fig. 6C). Loss of FTO also reduced the content of ATP and the number of mitochondria (Fig. 6D, E). Furthermore, si*Fto* eliminated the increased protein abundance of PGC1α in DHA-treated cells (Fig. 6F). Taken together, our results demonstrated that FTO mediated the positive regulation of DHA on PGC1α.

### FTO promotes PGC1α expression through DDIT4

**Fig. 7:**
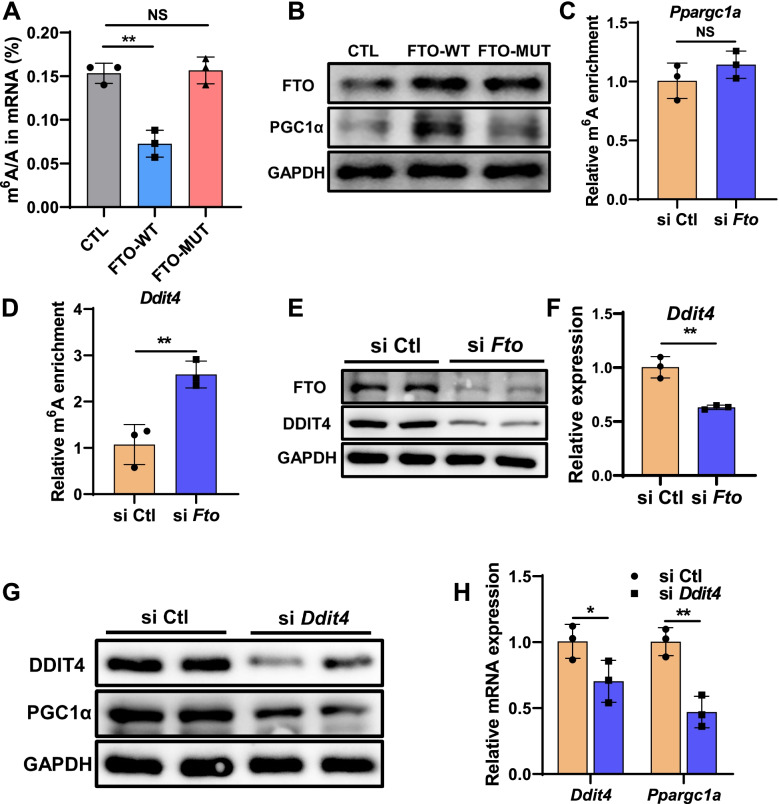
FTO promotes PGC1α expression through DDIT4. **A** LC-MS/MS quantification of the m^6^A/A in mRNA of control, FTO-WT, and FTO-MUT overexpressing cells (*n* = 3). **B** Western blot analysis of FTO and PGC1α expression in control, FTO-WT, and FTO-MUT overexpressing cells (*n* = 3). **C, D** Methylated RNA immunoprecipitation (MeRIP)-qPCR analysis of m^6^A levels of *Ppargc1a* and *Ddit4* mRNA in control and *Fto*-silencing cells (*n* = 3). **E** Western blotting analysis of FTO, DDIT4, and PGC1α in control and FTO knockdown cells (*n* = 3). **F** qPCR analysis of *Ddit4* in control and FTO knockdown cells (*n* = 3). **G** Western blotting analysis of DDIT4 and PGC1α in control and DDIT4 knockdown cells (*n* = 3). **H** qPCR analysis of *Ddit4* and *Ppargc1a* in control and DDIT4 knockdown cells (*n* = 3). Statistical analysis was performed using two-tailed paired Student’s *t* tests

Next question, we need to answer is how FTO regulates PGC1α. As we all known, FTO is one of the demethylases of m^6^A, which promotes us to test if the m^6^A demethylase activity of FTO is necessary for promoting PGC1α expression. We constructed FTO plasmid of wild-type (FTO-WT) and catalytic mutant FTOR96Q (FTO-MUT). The impact of FTO-WT or FTO-MUT on cellular m^6^A level was identified by LC-MS/MS (Fig. 7A), which proved their respective demethylase activity. Ectopically expressed FTO-WT, but not FTO-MUT nor an empty vector, significantly increased the protein expression of PGC1α (Fig. 7B), indicating that the expression modulation of FTO on PGC1α protein is demethylase activity-dependent. We then hypothesized that FTO regulated protein expression of PGC1α by increasing m^6^A levels of *Ppargc1a*. MeRIP-qPCR (m^6^A-IP-qPCR) was used to measure the relative m^6^A enrichment in *Ppargc1a* genes. Surprising, the relative m^6^A enrichment of *Ppargc1a* had no significant difference after *Fto* silence (Fig. 7C). Therefore, FTO did not directly regulate PGC1α expression through m^6^A, and we assumed that there should be another m^6^A target gene that mediates the effect. In combination with m^6^A sequencing data from previous studies, *Ddit4*, a potential upstream *Ppargc1a* gene, containing m^6^A modification was screened [37]. The result of MeRIP-qPCR showed that m^6^A enrichment of *Ddit4* mRNA increased after *Fto* interference (Fig. 7D), while WB and qPCR results showed that protein and mRNA expression levels of DDIT4 also decreased after interfering *Fto* with siRNA (Fig. 7E, F). In muscle tissue, HFD increased the m^6^A modification level of *Ddit4* mRNA and decreased its gene expression (Additional File 1: Fig. S3A, B). Similarly, FTO-WT can increase DDIT4 protein expression, while FTO-MUT does not seem to have this function (Additional File 1: Fig. S3C). These results suggest that *Ddit4* may be the direct target gene of FTO. To further identify whether DDIT4 mediated the regulation of FTO on PGC1α expression, DDIT4 was depleted in C2C12 myoblasts. We found that silencing of *Ddit4* inhibited the protein and mRNA expression of PGC1α (Fig. 7G, H). These data support that DDIT4 mediated the positive effect of FTO on PGC1α expression.

### YTHDF2 decreases DDIT4 protein expression by promoting mRNA decay

**Fig. 8:**
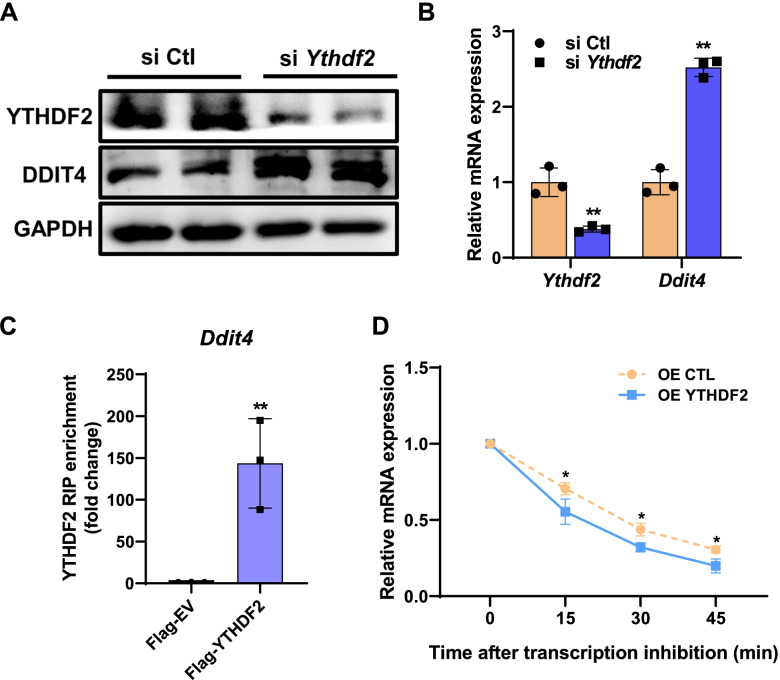
YTHDF2 affects DDIT4 protein expression by regulating mRNA stability. **A** Western blot analysis of YTHDF2 and DDIT4 in control and *Ythdf2* knockdown cells (*n* = 3). **B** qPCR analysis of *Ythdf2* and Ddit4 in control and *Ythdf2* knockdown cells (*n* = 3). **C** RIP-qPCR analysis of the interaction of *Ddit4* with YTHDF2 (*n* = 3). **D** Lifetime of *Ddit4* mRNA in control or YTHDF2 overexpression cells (*n* = 3). Relative mRNA levels were quantified by qPCR. Statistical analysis was performed using two-tailed paired Student’s *t* tests

**Fig. 9:**
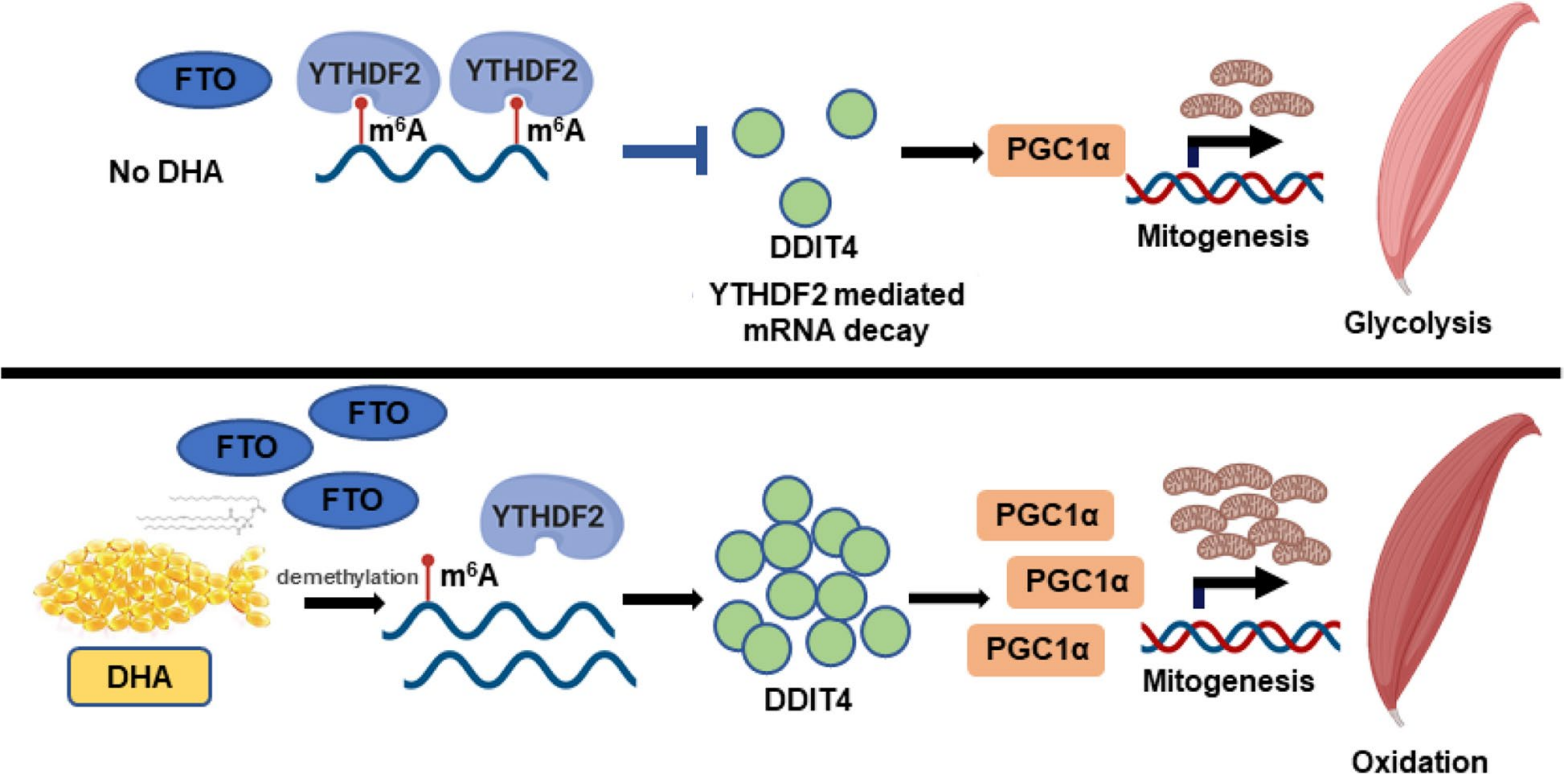
Working model of DHA in skeletal muscle remodeling

Our next question was how m^6^A affected the DDIT4 protein expression. Previous studies have shown that m^6^A modification exerts its function mainly depending on the recognition by specific m^6^A-binding proteins. In view of the negative correlation between m^6^A levels and protein expression of DDIT4, we assumed that YTHDF2 might mediate DDIT4 translation via binding m^6^A site of the transcript. Compared with control cells, silence of *Ythdf2* markedly promoted the protein and mRNA expression of DDIT4 (Fig. 8A, B). By RIP-qPCR assay, we further confirmed that *Ddit4* directly interacted with YTHDF2 (Fig. 8C). mRNA stability analysis showed that overexpression of YTHDF2 reduced the half-life of *Ddit4* (Fig. 8D). Consistently, results above suggest that YTHDF2 regulates DDIT4 expression via modulating mRNA stability Fig. 9.

## Discussion

Obesity can cause a decline in contractile function of skeletal muscle, thereby reducing mobility and promoting obesity-associated health risks. Dietary intervention is preferred as one of the most effective methods for improving skeletal muscle function, owing to its relatively easy operation and low costs. It has been found in previous studies the roles of various dietary nutrients including green tea, quercetin, curcumin, apigenin, DHA, and resveratrol in regulating skeletal muscle development, muscle mass, muscle function, and muscle recovery [38]. Other groups have previously documented a role for DHA in regulating the organ development and energy metabolism [39]. The goal of this research was to explore whether DHA could protect against obesity-induced dysfunction of skeletal muscle. In this study, we present several important advances in understanding the beneficial effects of DHA on skeletal muscle function. First, DHA improves skeletal muscle function by promoting muscle fiber type conversion from fast-twitch to slow-twitch in HFD. Second, DHA-induced muscle fiber type conversion is regulated by PGC1α-mediated mitochondrial biogenesis. Third, DHA increases the expression of PGC1α mediated by DDIT4, which is controlled by FTO. These data provide a mechanism whereby DHA alters muscle fiber type and affords protection by m^6^A modification during HFD.

Increasing evidence supports the beneficial effects of DHA on skeletal muscle function [18]. Several studies have reported that DHA-rich fish oil can modulate oxygen consumption during intense exercise and increase the efficiency of oxygen use in skeletal muscles [40, 41]. Other studies have shown that DHA supplementation can ameliorate endurance exercise capacity, which may be associated with increased expression of PGC1α in skeletal muscle that promotes mitochondrial biogenesis [15, 42]. However, there are some contradictions in the current reports on the effects of DHA on skeletal muscle under different physiological conditions, and its molecular mechanism needs to be further explored. At the same time, a growing body of evidence suggests that epigenetics is one of the mechanisms by which nutrients and bioactive compounds affect metabolic properties, also known as nutriepigenetic. DHA and its metabolites are also extensively involved in regulating epigenetic modifications. In some ways, DHA needs to be methylated to be metabolized further, so methyl groups need to be obtained directly or briefly from other methyl donors, such as S-adenosylmethionine (SAM)—the major methyl donors of m^6^A [43]. So theoretically, DHA may affect mRNA m^6^A modification to regulate the physiological functions. In this study, we provided convincing evidence that DHA markedly downregulated the level of m^6^A in skeletal muscle and myoblasts by promoting FTO expression in HFD.

RNA modifications, particularly the most abundant mRNA modification, m^6^A, have recently emerged as critical post-transcriptional regulators of gene expression programs. m^6^A modification affects almost every step in mRNA metabolism to some extent, including mRNA stability, splicing, and translation. The major mechanism by which m^6^A affects the fate of mRNAs is by recruiting m^6^A-binding proteins, in which YTHDF1 stabilize mRNA and promote translation [44], while YTHDF2 promotes degradation of m^6^A mRNAs by targeting them to P-bodies [45]. In our study, the negative correlation between m^6^A methylation and protein expression of DDIT4 indicates that YTHDF2 may mediate the degradation of DDIT4 mRNA. Our results demonstrated that DDIT4 mRNA directly binds to YTHDF2 and is degraded by YTHDF2.

To date, several studies have evaluated the effects of m^6^A modification on skeletal muscle and myoblasts. m^6^A modification and m^6^A-related proteins change dynamically during myogenic differentiation [24, 46]. METTL3 has been shown to be a regulator of muscle stem cell/myoblast state transitions in vitro and in vivo [24]. Depletion of m^6^A demethylase, FTO, led to impaired differentiation and fusion in vitro and impaired postnatal skeletal muscle development in vivo [21], and the overexpression of FTO promoted myoblast differentiation [22]. Similar to the results of our study, a dynamic pattern of FTO expression during myogenesis and different physiological processes in skeletal muscle has been observed [47, 48]; however, the upstream regulation of this expression remains elusive. A recent study on FTO has shown that multiple metabolites can bind to FTO and regulate m^6^A levels [35]. Since DHA can inhibit protein degradation [49], we speculate that DHA might directly bind to FTO to increase its protein stability. Further study is required to elucidate how DHA upregulates FTO expression. As regulators of methylation, betaine and cycloleucine are also involved in the regulation of m^6^A modification, thereby regulating various physiological processes [50, 51]. More recently, the dietary polyphenols, such as curcumin and epigallocatechin gallate, have been showed to exert bioactivity by modulating m^6^A modification in various tissues. Epigallocatechin gallate targeted FTO and inhibits adipogenesis in an m^6^A-dependent manner [32]. Overall, the current evidence strongly implies that diet and nutrition play a major role in m^6^A modification and interaction with metabolism, but the underlying mechanisms require further investigation.

At the same time, m^6^A has been shown to be responsive to a variety of dietary intervention. Several studies have investigated the relationship between high-fat diet and FTO; however, the expression of FTO in response to HFD appears to be different in various tissues, and its internal mechanisms are unclear. Although FTO was positively associated with fat deposition, FTO expression was decreased in white adipose tissue during HFD [52]. In the hypothalamus, FTO is downregulated in the hypothalamus of HFD compared to NFD rats [53]. The underlying mechanism of the effect of HFD on FTO level should be studied further.

Several studies have investigated the relationship between DDIT4 and mitochondria and found that DDIT4 can protect muscle by regulating mitochondrial-related metabolism under energy stress [54]. Whether and how DDIT4 influences mitochondrial biogenesis, however, remains controversial. Previous studies have shown that autophagy is necessary for mitochondrial biogenesis in skeletal muscle. While autophagic flux is blocked in skeletal muscle in vitro models of obesity, which may contribute to the decreases in mitochondrial content and function [55]. Since FTO and DDIT4 can positively regulate autophagy [56, 57], we speculate that DDIT4 might restore autophagy flux in skeletal muscle of obese mice, thereby promoting mitochondrial biogenesis. The mechanism of DDIT4 regulating PGC1α needs to be further studied.

In this study, we found that DHA promotes mitochondrial biogenesis and muscle fiber type conversion from fast-twitch to slow-twitch by downregulating m^6^A modification in skeletal muscle, predisposing mice to prevent the decline in skeletal muscle function induced by HFD. However, the molecular mechanism by which DHA regulates FTO protein expression remains to be further studied.

## Conclusions

In conclusion, we demonstrated that DHA reduced m^6^A level in skeletal muscle by elevating the expression of FTO, which promoted the transition of muscle fibers to oxidative fiber depending on its demethylase activity. We further uncovered FTO positively regulates PGC1α expression in an mRNA m^6^A-DDIT4-YTHDF2-dependent manner.

## Methods

### Animals

All experimental procedures were approved by the Committee on Animal Care and Use and Committee on the Ethics of Animal Experiments of Zhejiang University. Three-week-old male C57BL/6J mice (*n* = 8) used in this study were purchased from Laboratory Animal Center of Zhejiang University and allowed acclimatization for 1 week. Four-week-old mice were randomly divided into three groups. The mice then fed NFD (Jiangsu Xietong, XTCON50J, 10% calories from fat), HFD (Jiangsu Xietong, XTHF60, 60% kcal from fat), or HFD+DHA (Xi’an Tuofeng, purity quotient of 80%) for 10 weeks (Additional File 2: Table. S1). DHA was administered by oral gavage at a dose of 1 g/kg body weight, once in a day, which was based on the previous studies [42, 58]. Mice in NFD or HFD group received an equivalent dose of sterile phosphate buffer solution (PBS) by oral gavage as a control. Animals were maintained at constant temperature and humidity (22 ± 2 °C, 35 ± 10%) and kept on a 12-h light/dark cycle with free access to food and water. Body weight and food intake were monitored. At the end of the experiment, all mice were anesthetized with diethyl. Blood samples were collected, kept at 4 °C for overnights and centrifuged (4 °C, 3000 rpm, 20 min) to obtain the serum samples. Tissues were dissected out and either soaked into formalin for later histological analysis or soaked in liquid nitrogen and stored at − 80°C for further analysis.

### Skeletal muscle function test

To conduct the inverted screen test, mice were placed upright on a wire mesh screen that was subsequently turned 180°. The time when the mouse falls off would be recorded [59]. For the running distance test, mice were deprived of food for 2 h and acclimatized to the treadmill (NJKEWBIO, KW-PT) for 1 week at a gradual increase speed (5–10 m/min) for 10 min. Running distance tests were performed with 10° incline and mice ran at 10 m/min for the first 10 min followed by increases of 2 m/min every 10 min until exhaustion, which was defined by inability to remain on the treadmill for > 5 s (0.5 mA). Mice were removed immediately after exhaustion [60].

### Glucose tolerance test

The food was removed from mice for 6 h. Glucose tolerance test was performed by intraperitoneal injection with glucose at 1.5 g/kg body weight. Blood was collected from the tail vein and glucose levels were measured at the indicated times (0, 15, 30, 60, 90, and 120 min post-injection). Mice performed the previous bout of exercise test at least 12 h prior to start of tolerance test to avoid acute effects of exercise on glucose metabolism.

### Biochemical and histological analysis

The triglyceride assay kit (Applygen Technologies Inc, E1003) was used to determine serum triglyceride levels. In the triglyceride assay protocol, triglycerides are converted to free fatty acids and glycerol. Glycerol is then oxidized to generate a product which reacts with a probe to generate color and fluorescence. The muscle fibers were stained with ATPase staining method (Solarbio, G2380). In brief, gastrocnemius muscle from mice was frozen in isopentane near its freezing point and made into 10-μm-thick frozen sections. Frozen sections were then incubated in acid preincubation solution (pH 4.2, 5 min), ATPase incubation solution (30 min), 1% CaCl_2_ solution (3 min), cobalt solution (3 min), and sulfide working solution (1 min). The frozen sections were then washed with water and decolorized with anhydrous ethanol. Slow muscle fiber type was determined by the ATPase-positive part (black precipitation).

### Lipid analysis

#### Preparation of standards

The mixed standard solution of 40 fatty acid methyl esters was prepared into ten concentrations from 0.5 to 1000 mg/L. Then 25 μl of nonadecanoic acid methyl (500 ppm) is added into 500 μl of mixed standard as internal standard, mixed well, added into the injection bottle and enter Gas Chromatography-Mass Spectrometry (GC-MS) for detection.

#### Lipid extraction

Lipid extraction is done as follows: take 30 mg sample, successively add 1 ml chloroform methanol solution, ultrasonic for 30 min, take the supernatant, add 1% sulfuric acid methanol solution, esterificate in 80 °C water bath, extract with n-hexane, wash with pure water, absorb the supernatant, add nonadecanoic acid methyl as internal standard, mix well, and enter GC-MS for detection.

#### C-MS analysis

The samples were separated and analyzed by Agilent DB-WAX capillary column gas chromatography system and Agilent 7890/5975c gas chromatography-mass spectrometry.

#### Data analysis

Bioinformatic analysis was performed using the OmicStudio tools at https://www.omicstudio.cn/tool.

### Cell culture and DHA solution preparation

C2C12 myoblasts were maintained in culture at < 80% confluence in DMEM (Gibco, C11995500CP), 10% fetal bovine serum (Gibco, 10091-148), and 1% penicillin-streptomycin. To induce differentiation, media was changed from 10% FBS to 2% horse serum (Sangon Biotech, E510006-0100) when the cell density reaches 90%. DHA (Sigma, D2534) was dissolved in anhydrous ethanol and saponified by adding the preheated NaOH solution drop by drop. The DHA-NaOH solution was then added drop by drop to the preheated bovine serum albumin (BSA) free of fatty acid (Solarbio, A8850) solution in a molar ratio of 4:1 (DHA/BSA). The control solution contained equal amounts of NaOH and BSA. The DHA stock solution (2 mM) was filtered and sterilized by a 0.22-μm filter head (NEST Biotechnology, 331001), then separated and frozen. Cells were treated with DHA for 2 days or 4 days based on the purpose of experiment.

### Cell transfection with siRNA and plasmids

The siRNA and plasmids were transfected with lipofectamine RNAiMAX Transfection Reagent (Invitrogen, 13778150), lipofectamine 2000 Transfection Reagent (Invitrogen, 11668030), and EZ Trans (Life-iLab, China, AC04L091). The siRNA was ordered from GenePharma, and the sequences of siRNA are as follows (5′ to 3′). Mouse *Fto* siRNA: TTAAGGTCCACTTCATCATCGCAGG. Mouse *Ddit4* siRNA: AGGAAGAGGAGGACGAGAAACG.

The wild-type FTO-CDS expression plasmid was generated by cloning the full-length ORF of mouse *Fto* gene (NM_011936.2) into a pPB expression vector (Addgene, 48753). The mutant FTO R96Q-CDS was amplified by PCR and cloned into pPB vector. The FLAG-tagged mouse *Ythdf2* gene plasmids were cloned into a pPB vector.

### Western blot and immunofluorescence

Tissues and cells were solubilized in RIPA Lysis buffer. The supernatant protein lysates were collected after centrifugation (4 °Ci 12,000 rpm, 20 min) and separated by SDS-PAGE. For indirect immunofluorescence, cells were cultured on glass bottom dishes, fixed with 4% paraformaldehyde, and permeabilized with 0.1% Triton X-100. After blocking with 5% BSA for 1 h, the cells were then incubated with rabbit anti-MyH7 (1:500) at room temperature for 2 h, washed 3 times in PBS, and incubated with goat anti-rabbit Alexa 594 secondary antibody for 1 h at room temperature. Nuclei were stained with 1 μg/ml DAPI (US EVERBRIGHT, D4054) for 10 min at room temperature. The primary antibodies used in this study were described in Additional File 2: Table. S2. The gray value was analyzed by ImageJ.

### Gene expression

qRT-PCR was used to determined mRNA expression. Total RNA from myoblasts and skeletal muscle tissue were extracted using RNAiso Plus (Takara, 9109) and reverse transcribed into cDNA using M-MLV reverse transcriptase (Invitrogen, K1691). qPCR analysis was performed using the SYBR Green PCR Master Mix (Roche, 4913949001) with the ABI Step-One PlusTM Real-Time PCR System (Applied Biosystems). The expressions of target genes were then determined using the 2^−ΔΔCt^ method. The sequences of primers used in this study are presented in Additional File 2: Table. S3.

### Mitochondrial content assay

To quantify mitochondrial content, we tested mtDNA content and used Mito-Tracker Green probe (Beyotime, C1048). mtDNA was quantified via real-time fluorescent quantitative PCR (qRT-PCR) by measuring the ratio of *Cox2* gene to an intron of the *β-globin* gene. The Mito-Tracker Green probe was added to the cell culture medium and incubated at 37 °C for 30 min. Mito-tracker Green staining working solution was removed, and fresh cell culture solution preincubated at 37 °C was added. The mitochondrial staining was then observed with a fluorescence microscope.

### Oil Red O staining

Washed by PBS, myoblasts were fixed in 10% formalin at room temperature for 1 h. After washed with 60% isopropanol, the cells were stained with a filtered Oil Red O (Sigma-Aldrich, O0625). After rinsing with PBS, the lipid droplets dyed red were observed directly under the microscope. To quantify the relative lipid accumulation, Oil Red O-stained lipids were eluted in 100% isopropanol, and the optical density (OD) was measured at 500 nm.

### mRNA m^6^A quantification by LC-MS/MS or Dot Blot Analysis

To purify mRNA from total RNA preps, Dynabeads™ mRNA Purification Kit (Invitrogen, 61006) was used by following the manufacturer’s protocols. As previously described [56], the purified mRNA is digested by nuclease and alkaline phosphatase and then filtered by 0.22 μm. The total amount of m^6^A in RNA was measured using LC-MS/MS as previously reported [61]. The ratio of m^6^A modification in adenine was calculated based on the determined concentrations. Dot Blot Analysis of m^6^A refers to the published literature [62].

### Methylated RNA immunoprecipitation-qPCR (MeRIP-qPCR) analysis

MeRIP-qPCR analysis was conducted as previously reported [56]. Briefly, 2 μg total RNA was reserved as input for each sample, then fragmented RNA was incubated with anti-m^6^A antibody coupled to Dynabeads (Invitrogen, 10002D) in immunoprecipitation buffer at 4 °C for 2 h. mRNA with m^6^A modification was eluted from dynabeads and precipitated. m^6^A enrichment was determined by qPCR analysis. The calculation formula is as follows: ΔCT _Control_ = CT(IP)-CT(Input), ΔCT _Treatment_ = CT(IP)-CT(Input), m^6^A enrichment = 2^^(−(ΔCT Treatment −ΔCT Control))^ .

### RNA immunoprecipitation-qPCR (RIP-qPCR) analysis

RIP-qPCR analysis was conducted according to a reported method [63]. The protein of FLAG-YTHDF2 overexpressed in cells was precipitated by immunoprecipitation technology, and the RNA binding with YTHDF2 was isolated. The result was analyzed by qPCR analysis.

### mRNA stability analysis

Cells were treated with actinomycin D (Sigma-Aldrich, A9415) at 5 μg/ml to inhibit mRNA production. After incubation for indicated time points, the cells were collected and the associated mRNA expression was detected by qPCR analysis.

### Statistical analysis

All data were presented as the mean ± SD. GraphPad Prism software (version 8.0.2) was used for statistical analysis. The specific analysis method is shown in the figure legend. Significance was established at * *P* < 0.05 or ** *P* < 0.01.

## Supplementary Information

**Additional file 1: Figure S1.** Histomorphology and H&E sections of different types of muscles after DHA supplementation. (A) The gastrocnemius (GAS), tibialis anterior (TA), soleus (SOL) and extensor digitorum longus (EDL). (B) Hematoxylin and eosin–stained sections of GAS. Scale bar: 500 μm. **Figure S2.** Effects of different concentrations of DHA on C2C12 differentiation and m^6^A. (A) The proliferative activity of cells with different concentrations of DHA (compared with DHA 0 μM) (n=3). (B) Myotubule with high concentration DHA (50 or 100 Μm, 4d) treatment in Bright field and H&E staining. (C) Western blotting analysis of MyHC expression, (D) qPCR analysis of key genes, *Myod1* and *Myog* (n=3). (E) Oil Red O staining of DHA-treatment C2C12 after four days of differentiation. (F) Quantification of relative lipid accumulation (n=3). (G) qPCR analysis of genes associated with adipogenesis, including *Cebpα*, *Pparγ*, *Fabp4* after 48h of DHA treatment (n=3). (H) ORO staining and (I) quantification of relative lipid accumulation in C2C12 treated with low concentration of DHA (5 or 10 μM, 4d) (n=3). (J) qPCR analysis of *Myod1* and *Myog* (n=3). (K) Dot Blot Analysis of m^6^A. Statistical analysis was performed using one-way ANOVA (Figure S2D-G and I-J) and two-tailed paired Student’s t-tests (Figure S2A). **Figure S3.** DDIT4 is regulated by FTO mediated m^6^A. (A) qPCR analysis the expression of *Ddit4* in NFD, HFD and HFD+DHA group (n=6). (B) Methylated RNA immunoprecipitation (MeRIP)-qPCR analysis of m^6^A levels of *Ddit4* mRNA in NFD, HFD and HFD+DHA group (n=3). (C) Western blot analysis of DDIT4 expression in CTL, FTO-WT, FTO-MUT transfected C2C12 cells. Statistical analysis was performed using two-tailed paired Student’s t-tests.

**Additional file 2.** The lipid mass spec data of muscle after DHA supplementation.

**Additional file 3: Table S1.** NFD and HFD compositions. **Table S2.** The information of antibodies used in this study. **Table S3.** Primer sequences used in the qRT-PCR assays.

**Additional file 4.** Supporting data for the individual data values (n<6).

**Additional file 5.** The images of the original, uncropped gels/blots.

## Data Availability

All data generated or analyzed during this study are included in this published article, its supplementary information files and publicly available repositories. The lipid mass spec data generated in this article is available in Additional file 2. Feed composition, antibody, and primer information are included in Additional file 3. The individual data values (*n* < 6) are available in Additional file 4. The images of the original, uncropped gels/blots are available in Additional file 5.
