# Supplementary figures and images for: DHA alleviates diet-induced skeletal muscle fiber remodeling via FTO/m6A/DDIT4/PGC1α signaling
Source: BMC Biol. 2022 Feb 8;20:39. doi: 10.1186/s12915-022-01239-w (PMC8827147; doi:10.1186/s12915-022-01239-w)

**A**

**GAS TA SOL EDL**

**NFD**

**HFD**

**HFD+DHA**

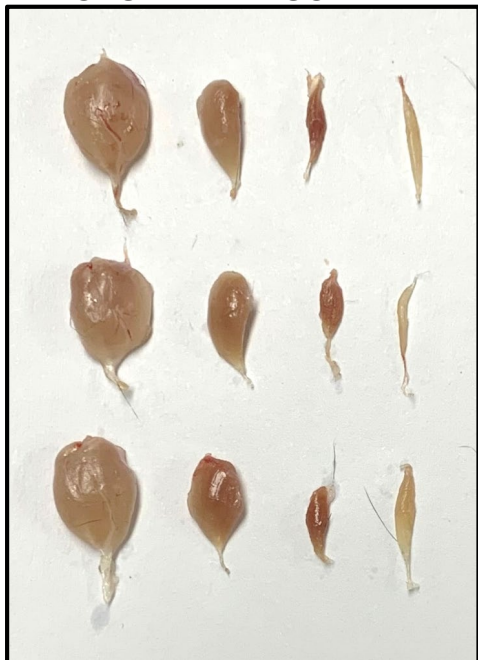

**B**

**NFD**

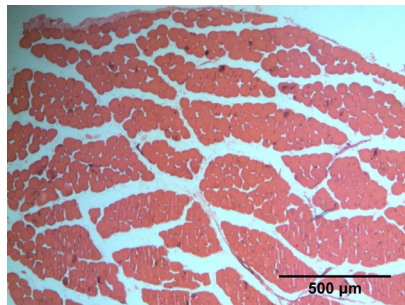

**HFD**

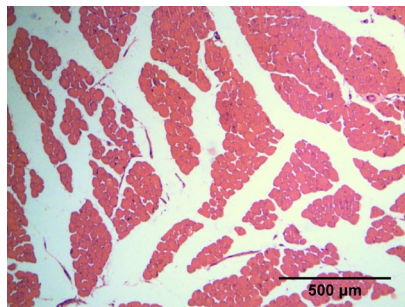

**HFD+DHA**

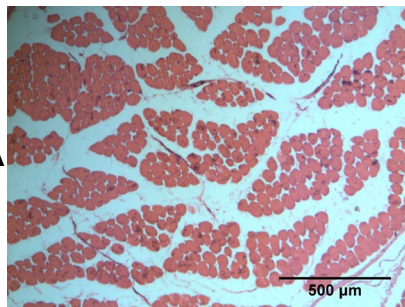

Supplement: Supplementary file 1 — Additional file 1: Figure S1. Histomorphology and H&E sections of different types of muscles after DHA supplementation. (A) The gastrocnemius (GAS), tibialis anterior (TA), soleus (SOL) and extensor digitorum longus (EDL). (B) Hematoxylin and eosin–stained sections of GAS. Scale bar: 500 μm. Figure S2. Effects of different concentrations of DHA on C2C12 differentiation and m6A. (A) The proliferative activity of cells with different concentrations of DHA (compared with DHA 0 μM) (n=3). (B) Myotubule with high concentration DHA (50 or 100 Μm, 4d) treatment in Bright field and H&E staining. (C) Western blotting analysis of MyHC expression, (D) qPCR analysis of key genes, Myod1 and Myog (n=3). (E) Oil Red O staining of DHA-treatment C2C12 after four days of differentiation. (F) Quantification of relative lipid accumulation (n=3). (G) qPCR analysis of genes associated with adipogenesis, including Cebpα, Pparγ, Fabp4 after 48h of DHA treatment (n=3). (H) ORO staining and (I) quantification of relative lipid accumulation in C2C12 treated with low concentration of DHA (5 or 10 μM, 4d) (n=3). (J) qPCR analysis of Myod1 and Myog (n=3). (K) Dot Blot Analysis of m6A. Statistical analysis was performed using one-way ANOVA (Figure S2D-G and I-J) and two-tailed paired Student’s t-tests (Figure S2A). Figure S3. DDIT4 is regulated by FTO mediated m6A. (A) qPCR analysis the expression of Ddit4 in NFD, HFD and HFD+DHA group (n=6). (B) Methylated RNA immunoprecipitation (MeRIP)-qPCR analysis of m6A levels of Ddit4 mRNA in NFD, HFD and HFD+DHA group (n=3). (C) Western blot analysis of DDIT4 expression in CTL, FTO-WT, FTO-MUT transfected C2C12 cells. Statistical analysis was performed using two-tailed paired Student’s t-tests. [file 12915_2022_1239_MOESM1_ESM.zip › Additional file 1 Fig. S1.pdf]

**A**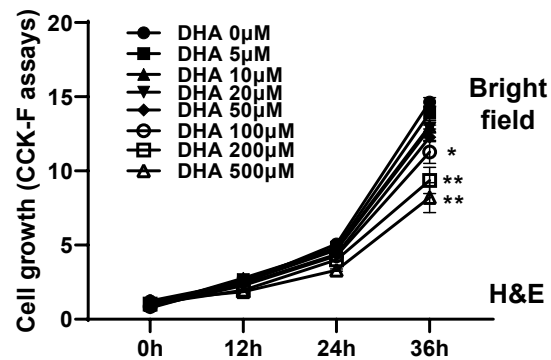**B**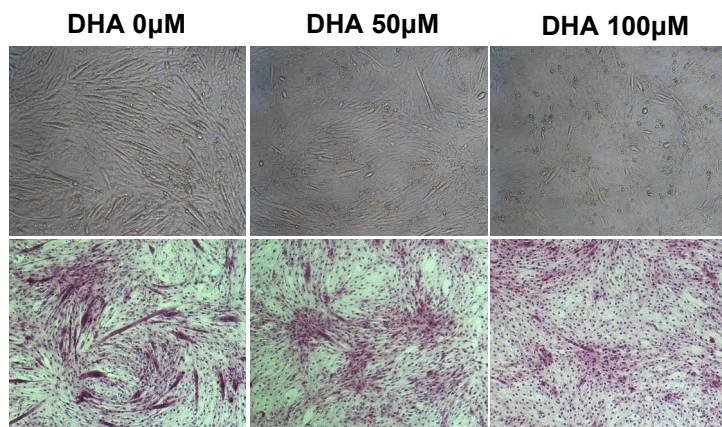**C**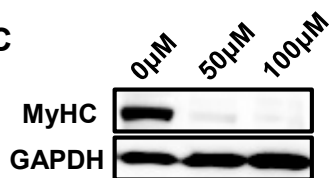**E**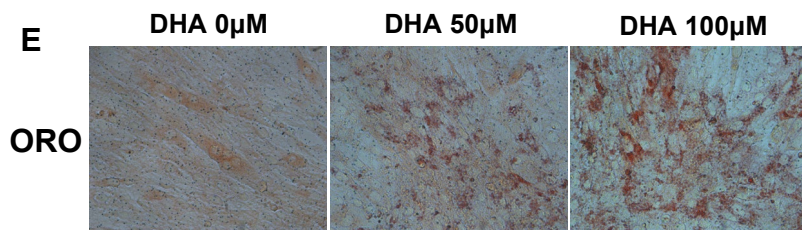**D**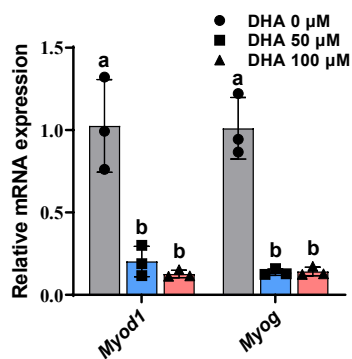**F**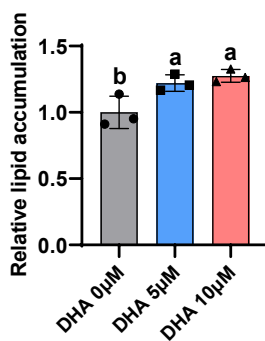**G**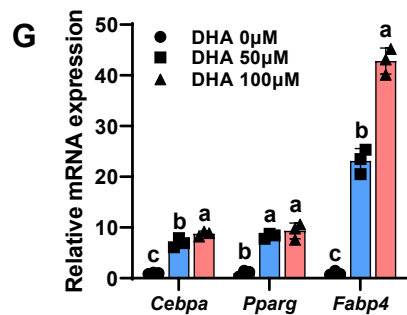**H**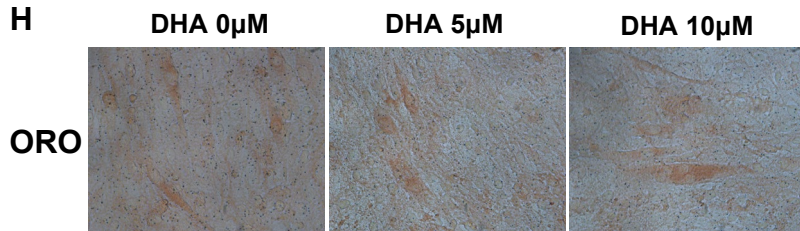**J**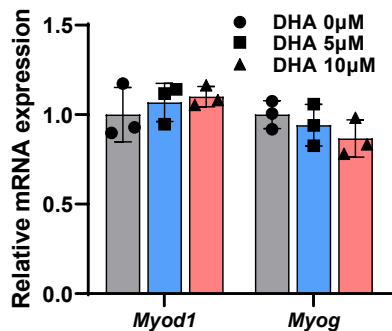**K**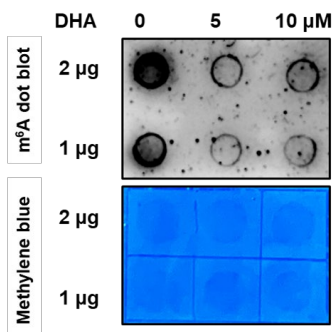**I**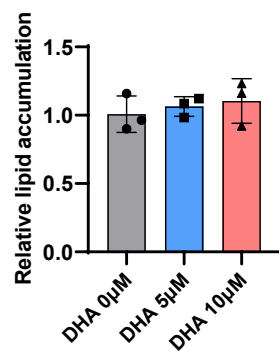

Supplement: Supplementary file 1 — Additional file 1: Figure S1. Histomorphology and H&E sections of different types of muscles after DHA supplementation. (A) The gastrocnemius (GAS), tibialis anterior (TA), soleus (SOL) and extensor digitorum longus (EDL). (B) Hematoxylin and eosin–stained sections of GAS. Scale bar: 500 μm. Figure S2. Effects of different concentrations of DHA on C2C12 differentiation and m6A. (A) The proliferative activity of cells with different concentrations of DHA (compared with DHA 0 μM) (n=3). (B) Myotubule with high concentration DHA (50 or 100 Μm, 4d) treatment in Bright field and H&E staining. (C) Western blotting analysis of MyHC expression, (D) qPCR analysis of key genes, Myod1 and Myog (n=3). (E) Oil Red O staining of DHA-treatment C2C12 after four days of differentiation. (F) Quantification of relative lipid accumulation (n=3). (G) qPCR analysis of genes associated with adipogenesis, including Cebpα, Pparγ, Fabp4 after 48h of DHA treatment (n=3). (H) ORO staining and (I) quantification of relative lipid accumulation in C2C12 treated with low concentration of DHA (5 or 10 μM, 4d) (n=3). (J) qPCR analysis of Myod1 and Myog (n=3). (K) Dot Blot Analysis of m6A. Statistical analysis was performed using one-way ANOVA (Figure S2D-G and I-J) and two-tailed paired Student’s t-tests (Figure S2A). Figure S3. DDIT4 is regulated by FTO mediated m6A. (A) qPCR analysis the expression of Ddit4 in NFD, HFD and HFD+DHA group (n=6). (B) Methylated RNA immunoprecipitation (MeRIP)-qPCR analysis of m6A levels of Ddit4 mRNA in NFD, HFD and HFD+DHA group (n=3). (C) Western blot analysis of DDIT4 expression in CTL, FTO-WT, FTO-MUT transfected C2C12 cells. Statistical analysis was performed using two-tailed paired Student’s t-tests. [file 12915_2022_1239_MOESM1_ESM.zip › Additional file 1 Fig. S2.pdf]

**A**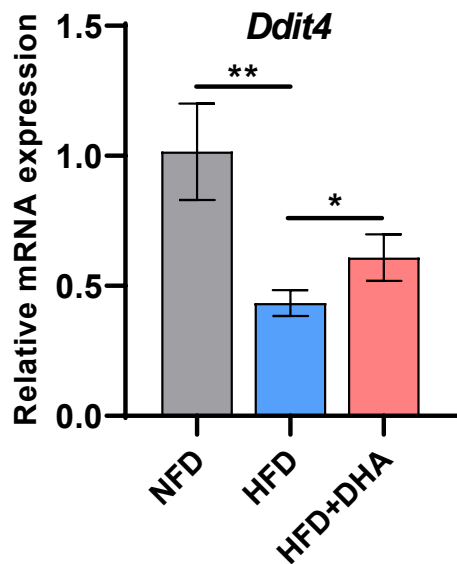**B**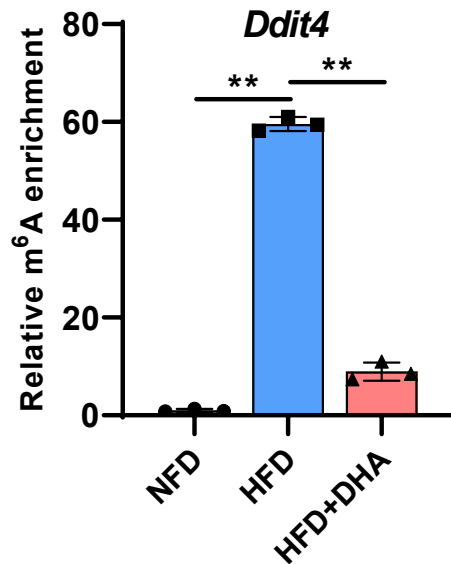**C**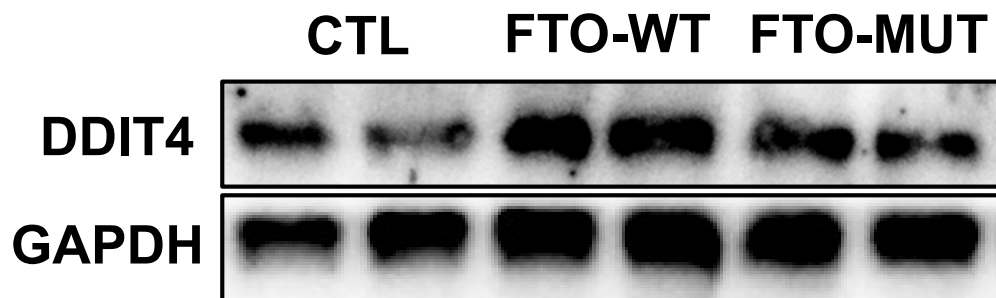

Supplement: Supplementary file 1 — Additional file 1: Figure S1. Histomorphology and H&E sections of different types of muscles after DHA supplementation. (A) The gastrocnemius (GAS), tibialis anterior (TA), soleus (SOL) and extensor digitorum longus (EDL). (B) Hematoxylin and eosin–stained sections of GAS. Scale bar: 500 μm. Figure S2. Effects of different concentrations of DHA on C2C12 differentiation and m6A. (A) The proliferative activity of cells with different concentrations of DHA (compared with DHA 0 μM) (n=3). (B) Myotubule with high concentration DHA (50 or 100 Μm, 4d) treatment in Bright field and H&E staining. (C) Western blotting analysis of MyHC expression, (D) qPCR analysis of key genes, Myod1 and Myog (n=3). (E) Oil Red O staining of DHA-treatment C2C12 after four days of differentiation. (F) Quantification of relative lipid accumulation (n=3). (G) qPCR analysis of genes associated with adipogenesis, including Cebpα, Pparγ, Fabp4 after 48h of DHA treatment (n=3). (H) ORO staining and (I) quantification of relative lipid accumulation in C2C12 treated with low concentration of DHA (5 or 10 μM, 4d) (n=3). (J) qPCR analysis of Myod1 and Myog (n=3). (K) Dot Blot Analysis of m6A. Statistical analysis was performed using one-way ANOVA (Figure S2D-G and I-J) and two-tailed paired Student’s t-tests (Figure S2A). Figure S3. DDIT4 is regulated by FTO mediated m6A. (A) qPCR analysis the expression of Ddit4 in NFD, HFD and HFD+DHA group (n=6). (B) Methylated RNA immunoprecipitation (MeRIP)-qPCR analysis of m6A levels of Ddit4 mRNA in NFD, HFD and HFD+DHA group (n=3). (C) Western blot analysis of DDIT4 expression in CTL, FTO-WT, FTO-MUT transfected C2C12 cells. Statistical analysis was performed using two-tailed paired Student’s t-tests. [file 12915_2022_1239_MOESM1_ESM.zip › Additional file 1 Fig. S3.pdf]
