# Supplementary material for: DHA alleviates diet-induced skeletal muscle fiber remodeling via FTO/m6A/DDIT4/PGC1α signaling
Source: BMC Biol. 2022 Feb 8;20:39. doi: 10.1186/s12915-022-01239-w (PMC8827147; doi:10.1186/s12915-022-01239-w)
Supplement: Supplementary file 3 — Additional file 3: Table S1. NFD and HFD compositions. Table S2. The information of antibodies used in this study. Table S3. Primer sequences used in the qRT-PCR assays. [file 12915_2022_1239_MOESM3_ESM.docx]

**Table S1.** NFD and HFD compositions.

| **Product** | **XTCON50J** | | **XTHF60** | |
| --- | --- | --- | --- | --- |
| **Ingredient** | **gm** | **kcal** | **gm** | **kcal** |
| **Casein, 80Mesh** | 189.57 | 758.29 | 258.46 | 1033.86 |
| **L-Cystine** | 2.84 | 11.37 | 3.88 | 15.51 |
| **Corn Starch** | 479.81 | 1919.24 | 0 | 0 |
| **Maltodextrin 10** | 118.48 | 473.93 | 161.54 | 646.16 |
| **Sucrose** | 65.21 | 260.85 | 88.91 | 355.65 |
| **Cellulose, BW200** | 47.39 | 0 | 64.62 | 0 |
| **Soybean Oil** | 23.7 | 213.27 | 32.31 | 290.77 |
| **Lard** | 18.96 | 170.62 | 316.62 | 2849.57 |
| **Mineral Mix** | 9.48 | 0 | 12.92 | 0 |
| **DiCalcium Phosphate** | 12.32 | 0 | 16.8 | 0 |
| **Calcium Carbonate** | 5.21 | 0 | 7.11 | 0 |
| **Potassium Citrate, 1 H_2_O** | 15.64 | 0 | 21.32 | 0 |
| **Vitamin Mix** | 9.48 | 37.91 | 12.92 | 51.69 |
| **Choline Bitartrate** | 1.9 | 0 | 2.58 | 0 |
| **FD&C Yellow Dye #5** | 0.04 | 0 | 0 | 0 |
| **FD&C Blue Dye #1** | 0.01 | 0 | 0.05 | 0 |
| **Total** | 1000 | 3845.49763 | 1000 | 5243.22 |

The feeds used in this experiment were all from Jiangsu Xietong Pharmaceutical Bio-engineering Co., Ltd.

XTCON50J, normal fat diet, is made up of 19.2% protein, 67.3% carbohydrate and 4.3% fat, 10% kcal from fat;

XTHF60, high fat diet ,is made up of 26% protein, 26% carbohydrate and 35% fat, 60% kcal from fat.

**Table S2.** The information of antibodies used in this study.

| **Antibodies** | **Source** | **Identifier** | **Host** | **Antibody dilutions** |
| --- | --- | --- | --- | --- |
| FTO | Abcam | ab126605 | Rabbit | 1:1000 (WB) |
| ALKBH5 | Proteintech | 16837-1-AP | Rabbit | 1:3000 (WB) |
| METTL3 | Proteintech | 15073-1-AP | Rabbit | 1:3000 (WB) |
| YTHDF2 | Millipore | ABE542 | Rabbit | 1:2000 (WB) |
| PGC1α | Abcam | Ab54481 | Rabbit | 1:1000 (WB) |
| DDIT4 | Proteintech | 10638-1-AP | Rabbit | 1:2000 (WB) |
| MyHC | Sigma | M7523 | Rabbit | 1: 1000 (WB) |
| slow MyHC | Sigma | M8421 | Mouse | 1:500 (IF) |
| GAPDH | HUABIO | ER1706-83 | Rabbit | 1:5000 (WB) |
|  |  |  |  |  |

**Table S3.** Primer sequences used in the qRT-PCR assays.

| **Genes** | **Forward Primer** | **Reverse Primer** |
| --- | --- | --- |
| *Gapdh* | 5’-AGGGCATCTTGGGCTACAC-3’ | 5’-TGGTCCAGGGTTTCTTACTCC-3’ |
| *Myh1* | 5’-CAACCCATACGACTACGCCT-3’ | 5’-CATCAGAAGTGAAGCCCAGAAT-3’ |
| *Myh2* | 5’-TTCCAGAAGCCTAAGGTGGTC-3’ | 5’-GCCAGCCAGTGATGTTGTAAT-3’ |
| *Myh4* | 5’-CTTGTCTGACTCAAGCCTGCC-3’ | 5’-TCGCTCCTTTTCAGACTTCCG-3’ |
| *Myh7* | 5’-CTTCTACAGGCCTGGGCTTAC-3’ | 5’-CTCCTTCTCAGACTTCCGCAG-3’ |
| *Tnni1* | 5’-GATGGGAGATGAGGAGAAGC-3’ | 5’-GGGCAGTGTTCTGACAGGTA-3’ |
| *Tnni2* | 5’-GGCCTCTGAGCACAGAAAGT-3’ | 5’-GCCGGGAGCTATCTTTCTTA-3’ |
| *Mb* | 5’-GGAAGTCCTCATCGGTCTGT-3’ | 5’-GGTCCTCTGAGCCCTTCATA-3’ |
| *Myod1* | 5’-CCACTCCGGGACATAGACTTG-3’ | 5’-AAAAGCGCAGGTCTGGTGAG-3’ |
| *Myog* | 5’-CCTGGAAGAAAAGGGACTGG-3’ | 5’-CGCTCAATGTACTGGATGGC-3’ |
| *Cox2* | 5’-GCCGACTAAATCAAGCAACA-3’ | 5’-CAATGGGCATAAAGCTATGG-3’ |
| *β-globin* | 5’-GAAGCGATTCTAGGGAGCAG-3’ | 5’-GGAGCAGCGATTCTGAGTAGA-3’ |
| *Cebpa* | 5’-GGTTTCGGGTCGCTGGATCTCTAG-3’ | 5’-ACGGCCTGACTCCCTCATCTTAGAC-3’ |
| *Pparg* | 5’-TGGGTGAAACTCTGGGAGATTC-3’ | 5’-AGAGGTCCACAGAGCTGATTCC-3’ |
| *Fabp4* | 5’-GTGTGATGCCTTTGTGGGAAC-3’ | 5’-CCTGTCGTCTGCGGTGATT-3’ |
| *Fto* | 5’-GAGCAGCCTACAACGTGACT-3’ | 5’-GAAGCTGGACTCGTCCTCAC-3’ |
| *Ythdf2* | 5’-CAGGCAAGGCCGAATAATGC-3’ | 5’-TCTCCGTTGCTCAGTTGTCC-3’ |
| *Ppargc1a* | 5’-GAGCCATTTCTTCCACAATCCA-3’ | 5’-TCTCCCATTCAATCCTTACGCT-3’ |
| *Ddit4* | 5’-AGGAAGAGGAGGACGAGAAACG-3’ | 5’-CGCCTTCATTCGGACCTTAG-3’ |
