# Supplementary material for: DHA alleviates diet-induced skeletal muscle fiber remodeling via FTO/m6A/DDIT4/PGC1α signaling
Source: BMC Biol. 2022 Feb 8;20:39. doi: 10.1186/s12915-022-01239-w (PMC8827147; doi:10.1186/s12915-022-01239-w)
Supplement: Supplementary file 5 — Additional file 5. The images of the original, uncropped gels/blots. [file 12915_2022_1239_MOESM5_ESM.pptx]

## Slide 1
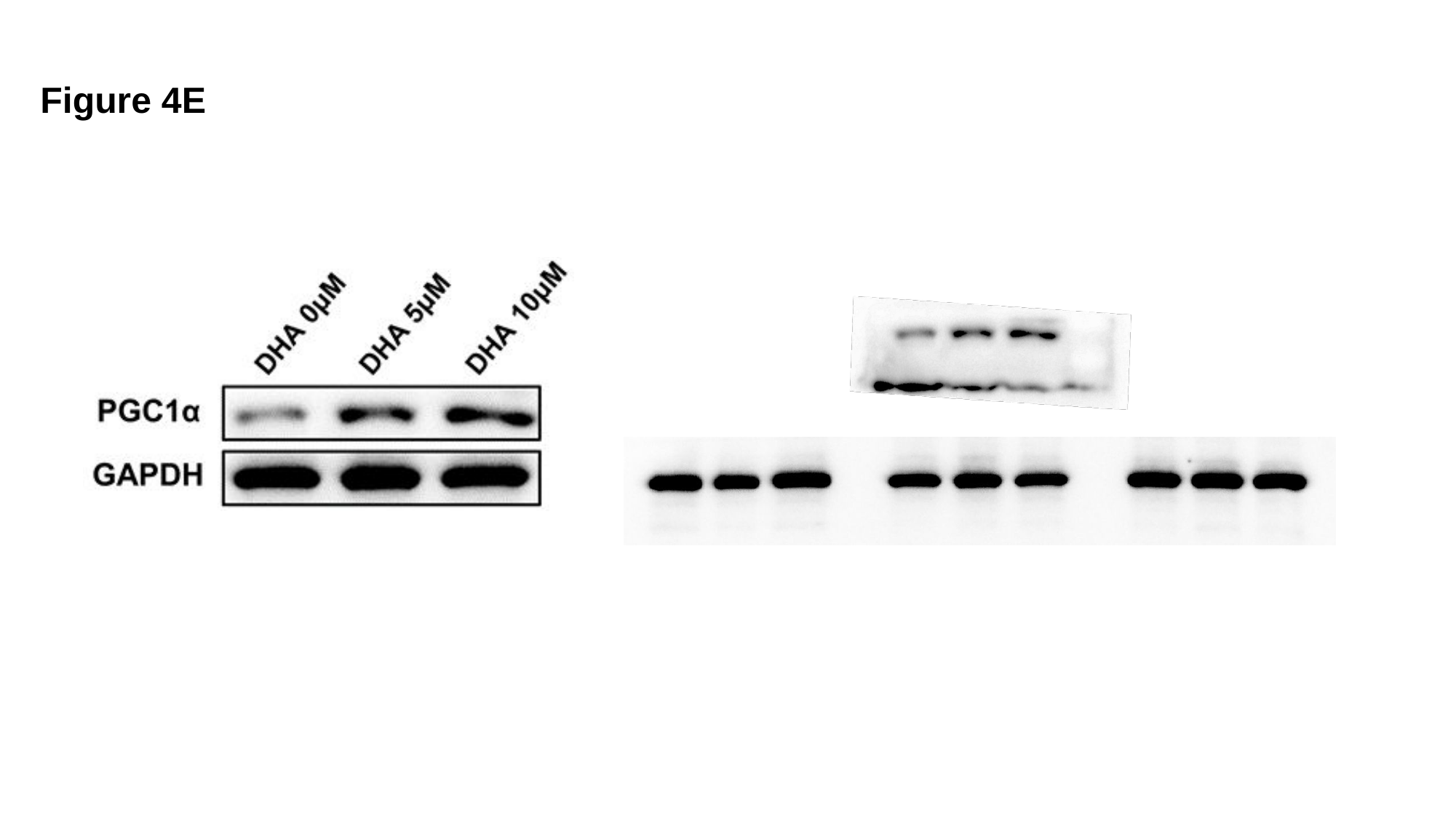

Figure 4E

## Slide 2
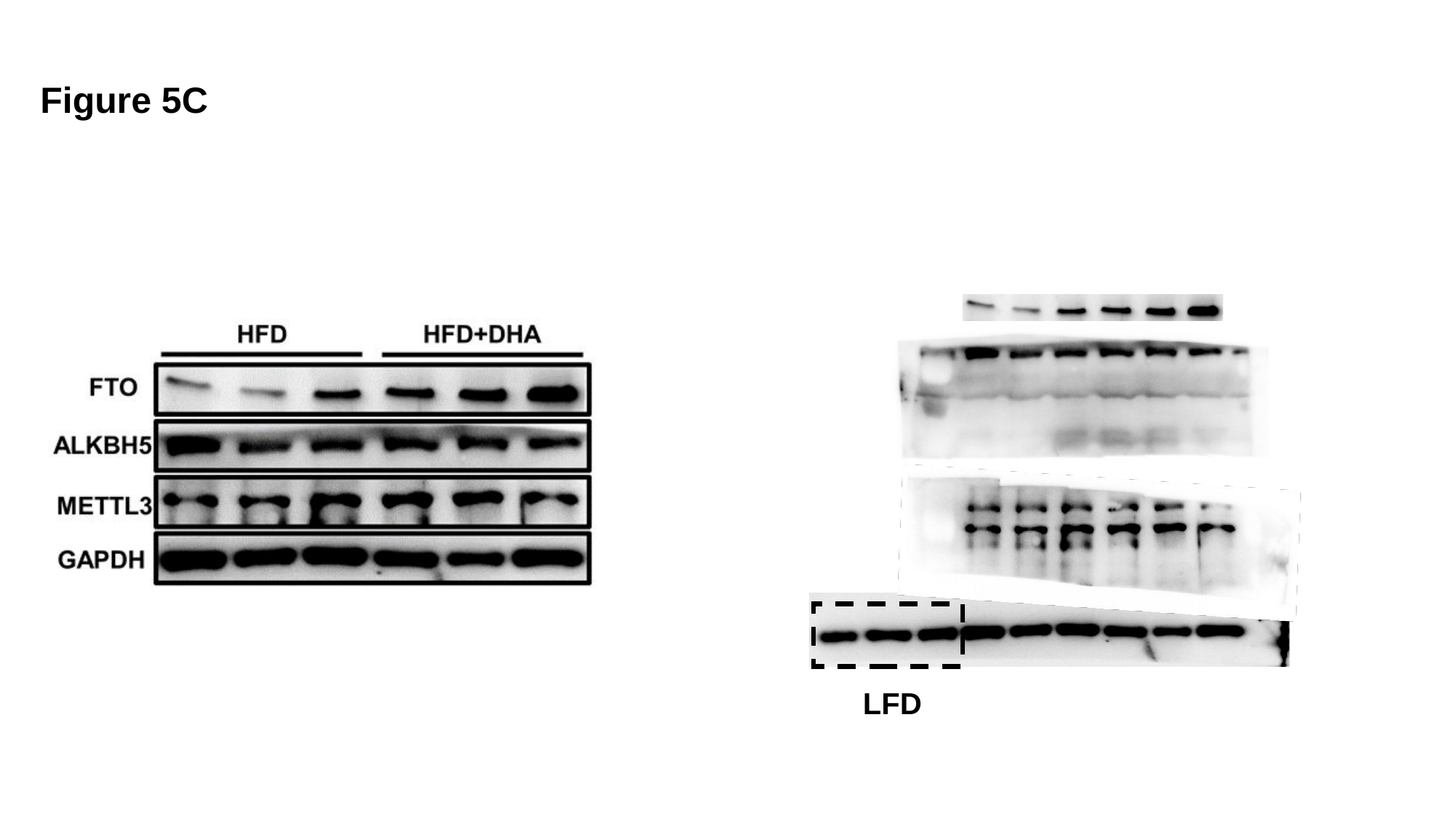

Figure 5C
LFD

## Slide 3
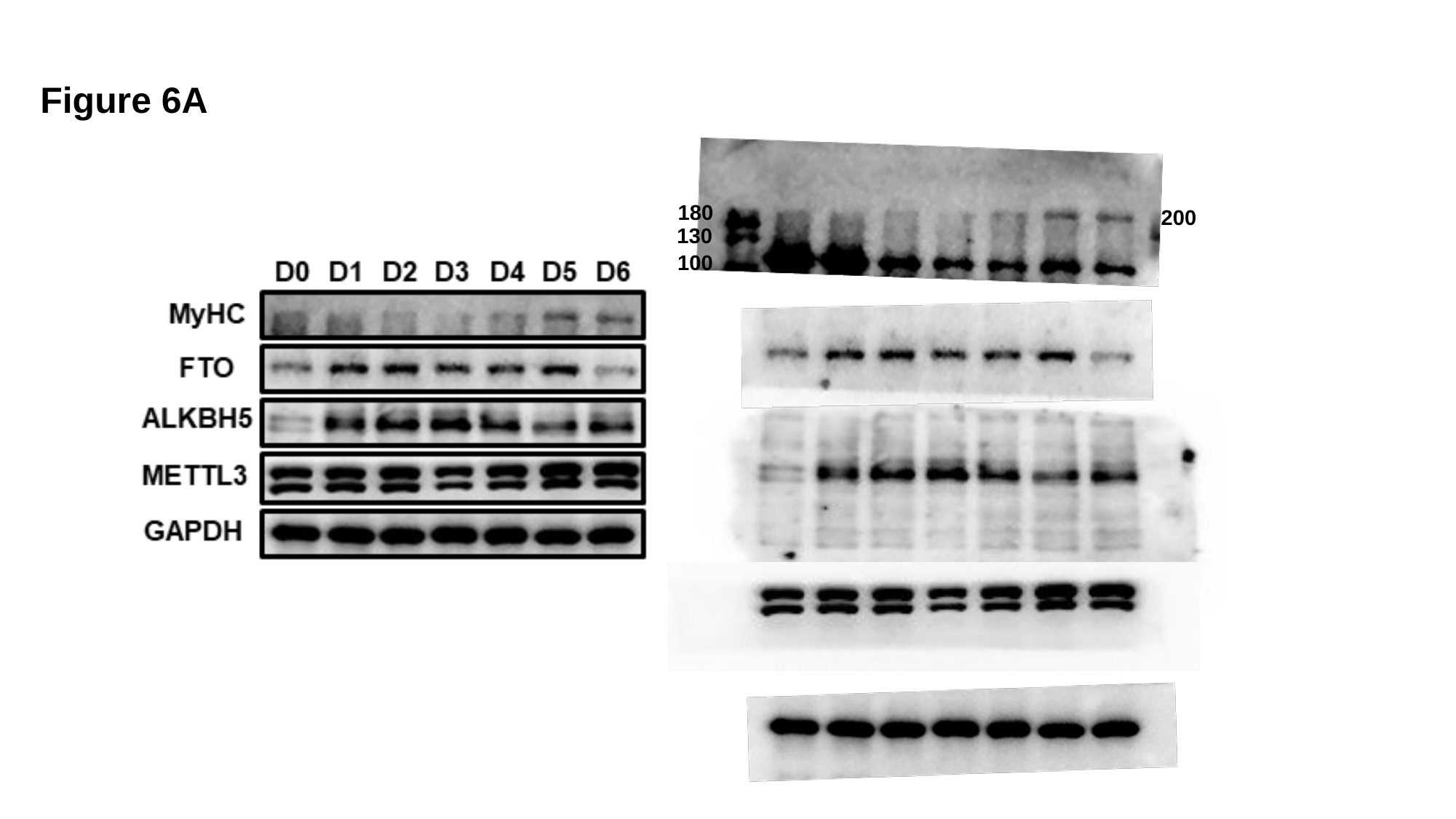

MyHC
Figure 6A
180
200
130
100

## Slide 4
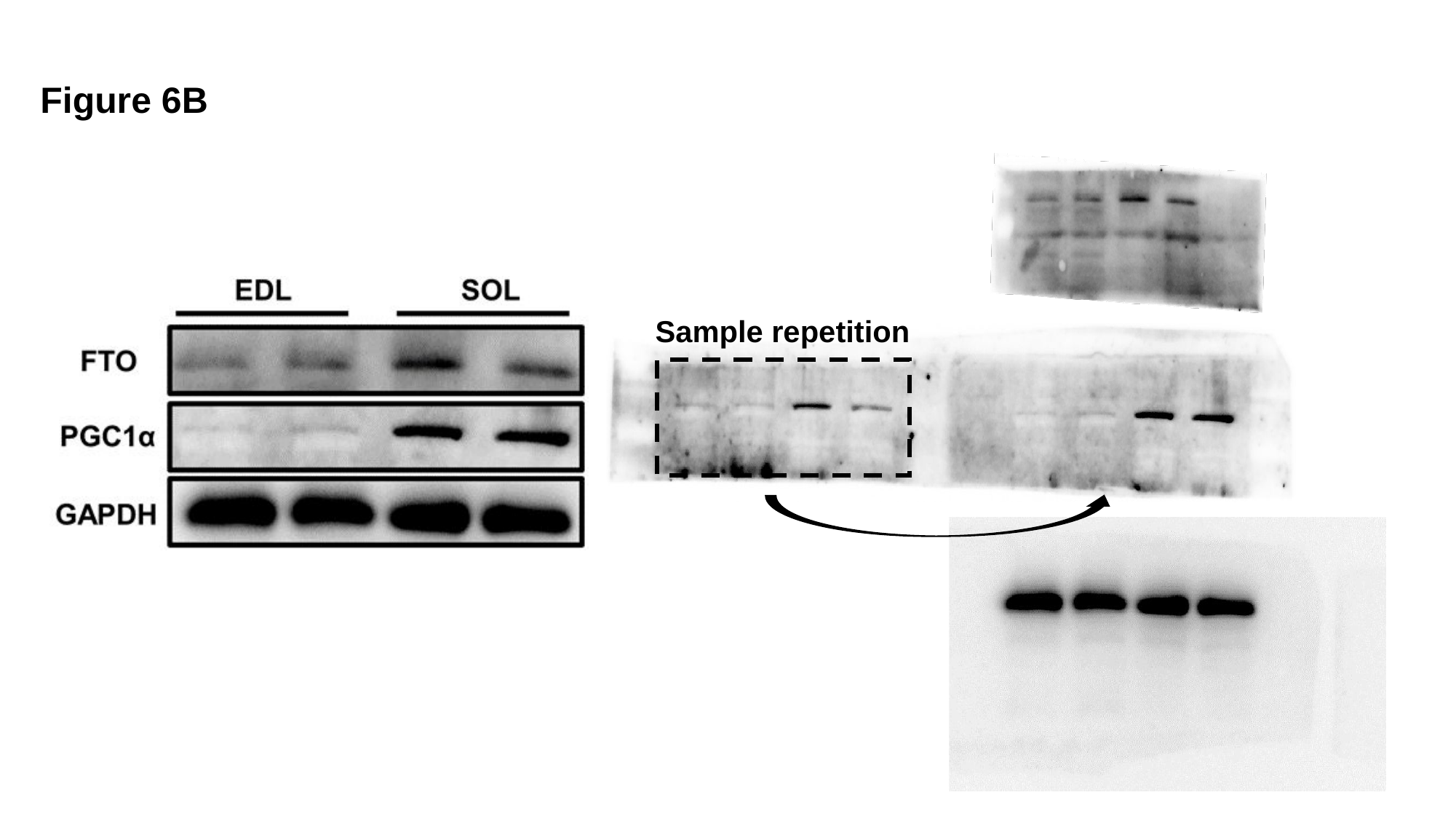

Figure 6B
Sample repetition

## Slide 5
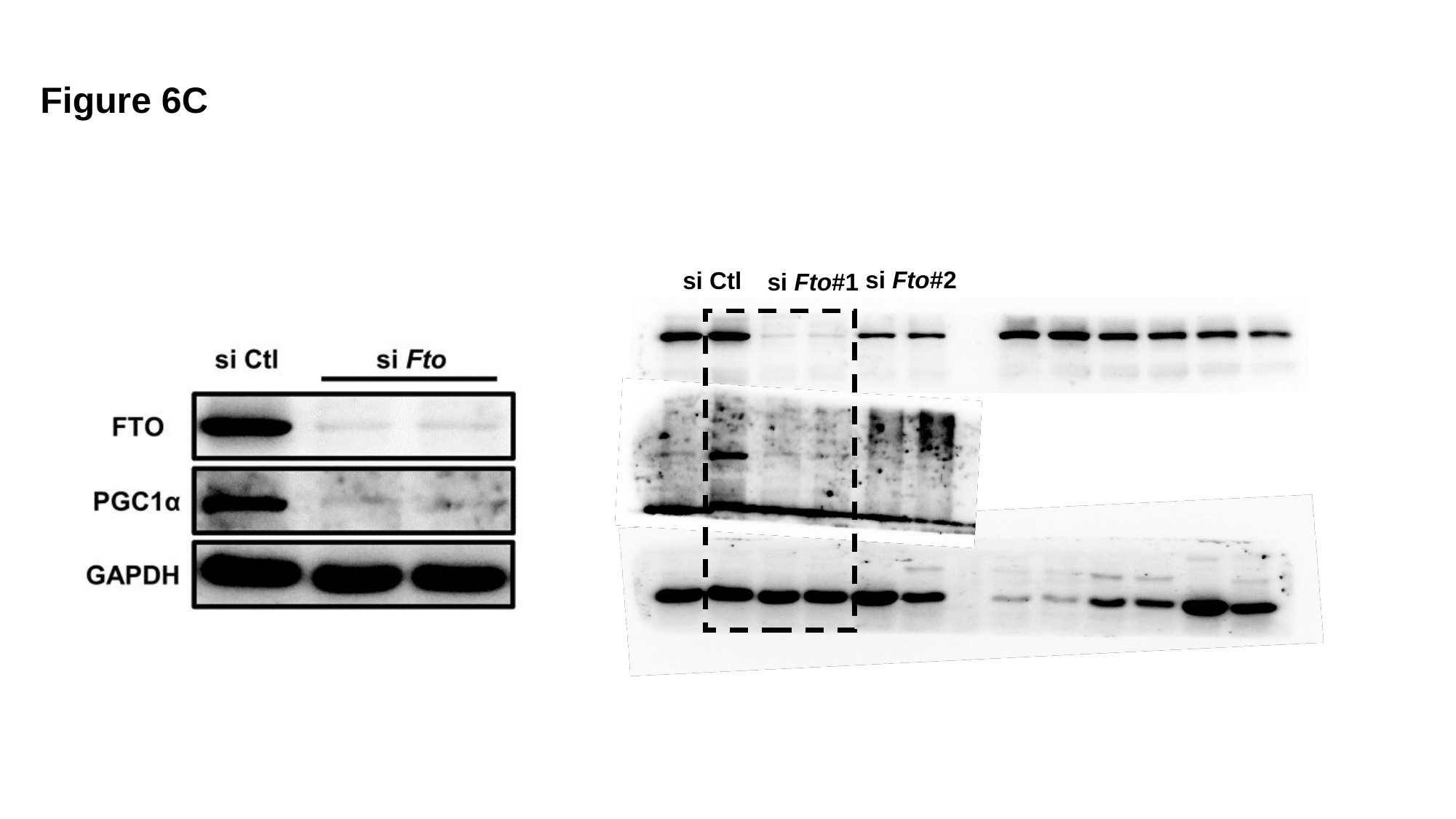

Figure 6C
si Fto#2
si Ctl
si Fto#1

## Slide 6
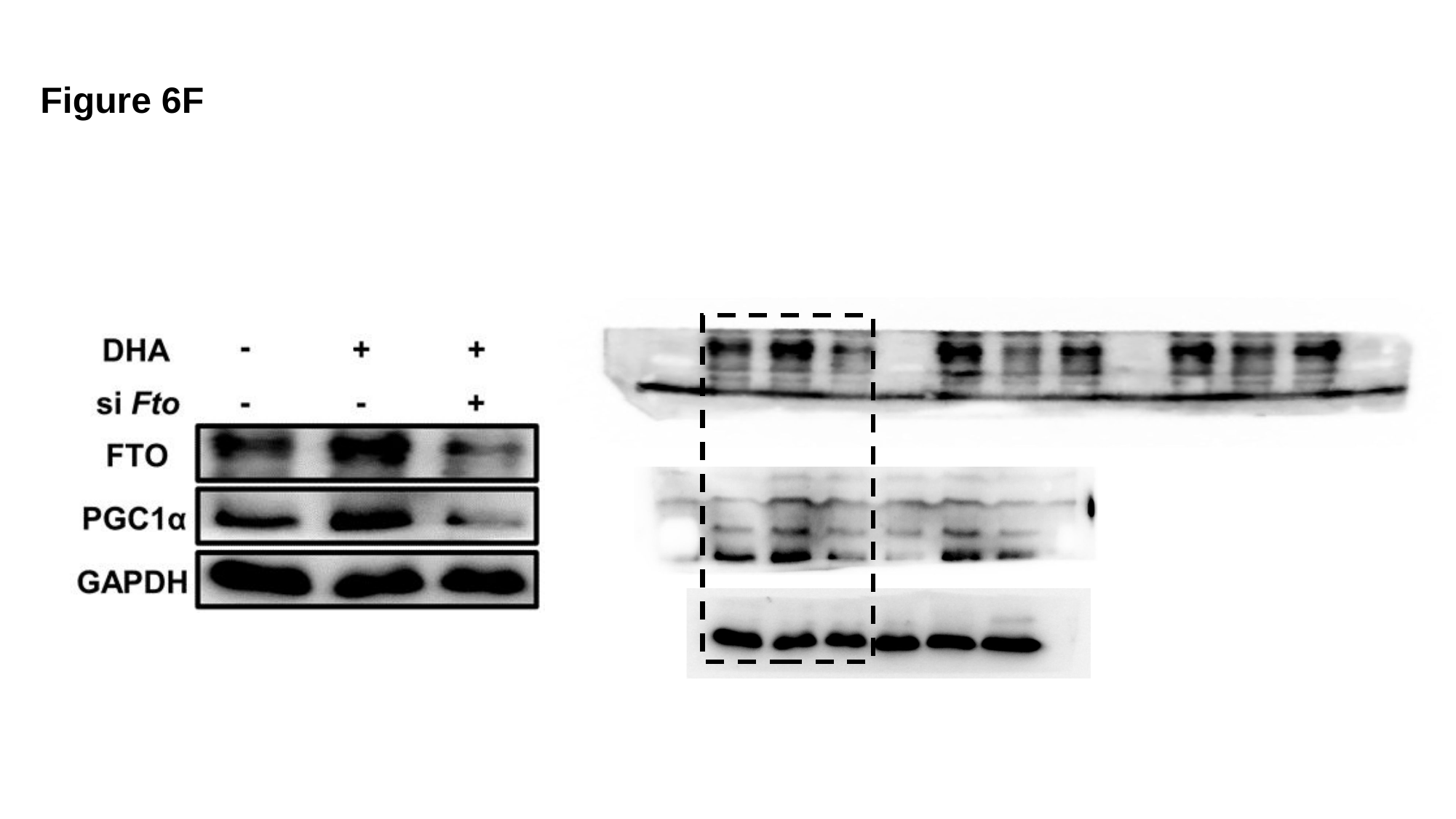

Figure 6F

## Slide 7
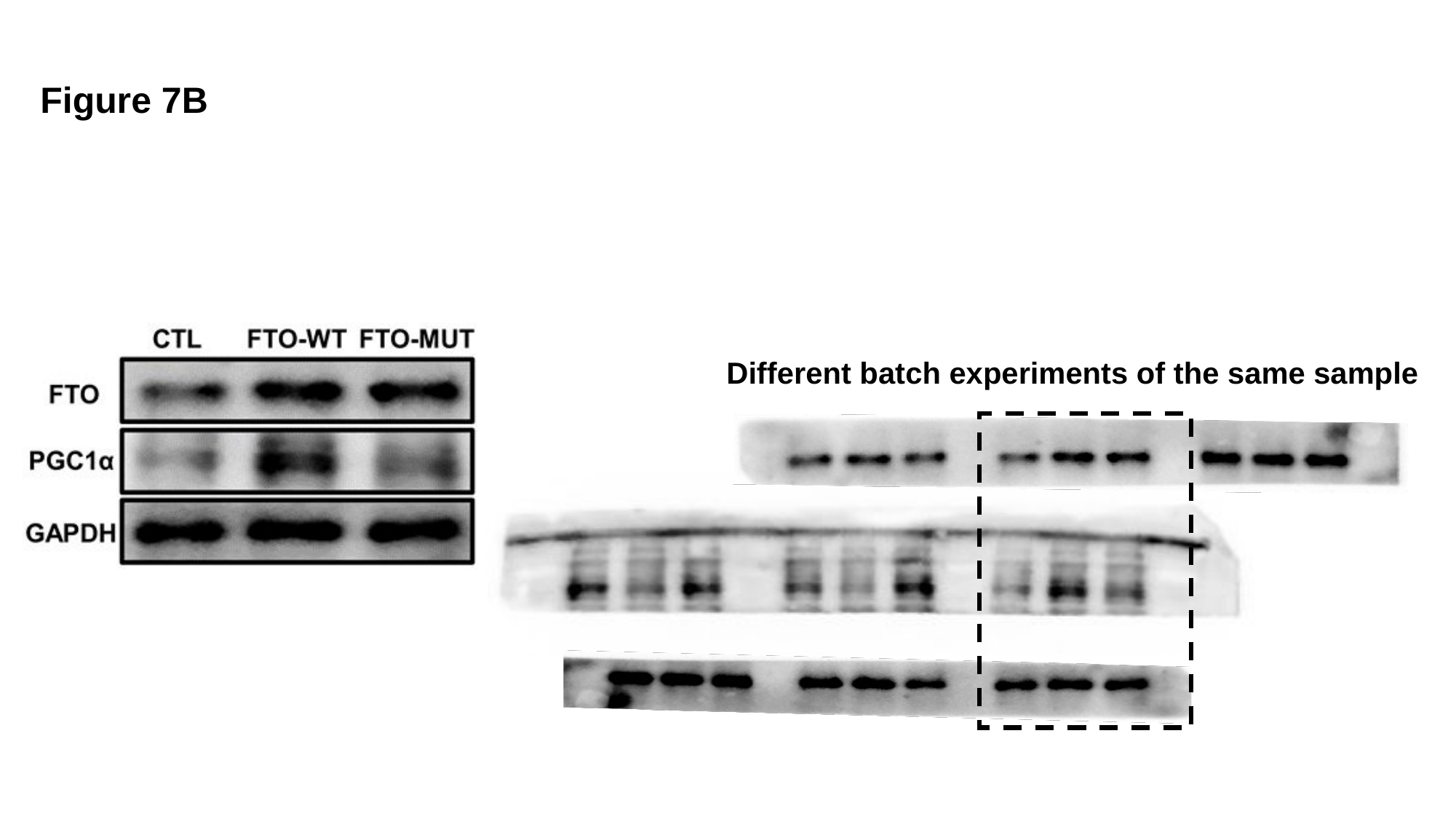

Figure 7B
Different batch experiments of the same sample

## Slide 8
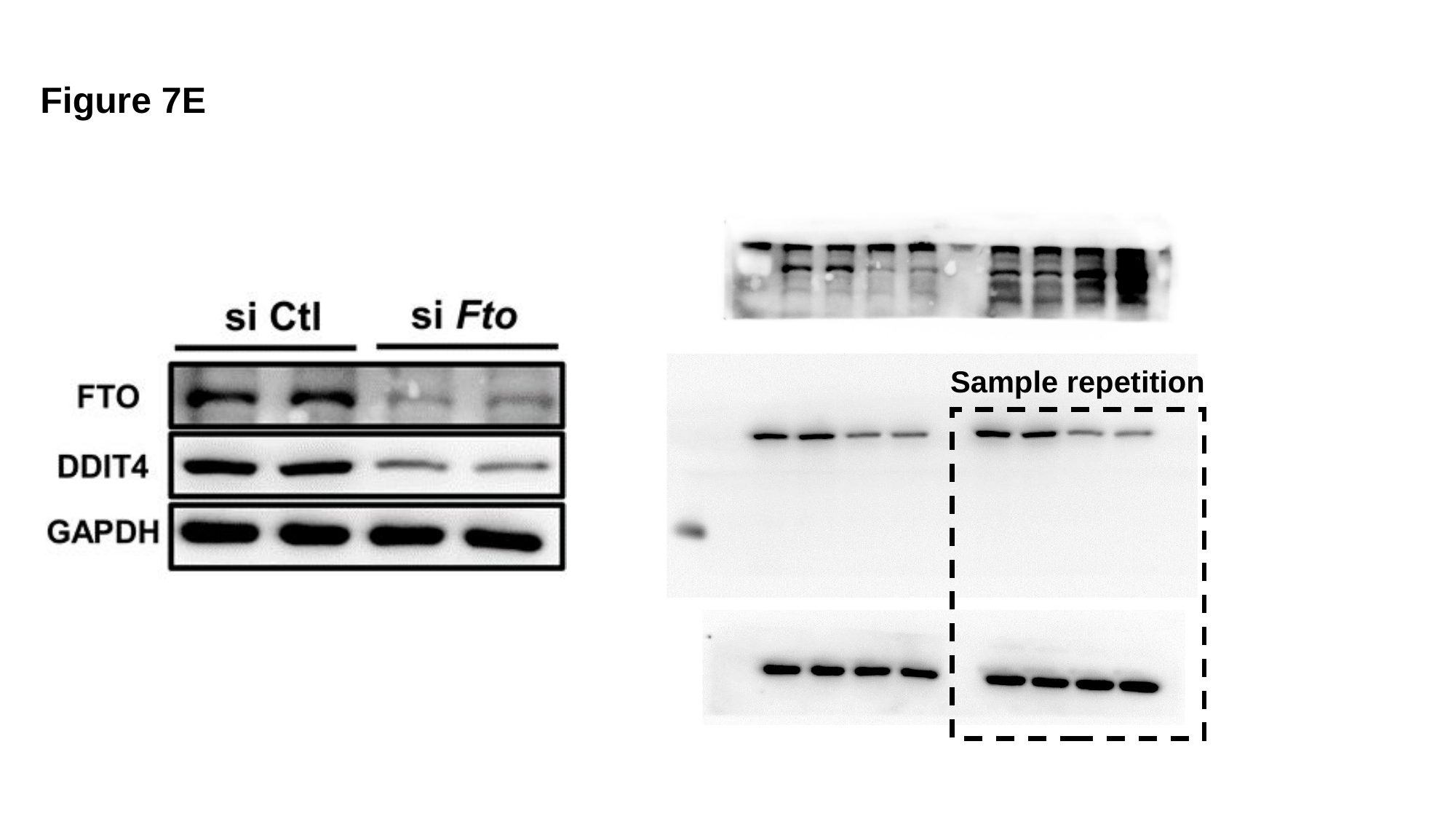

Figure 7E
Sample repetition

## Slide 9
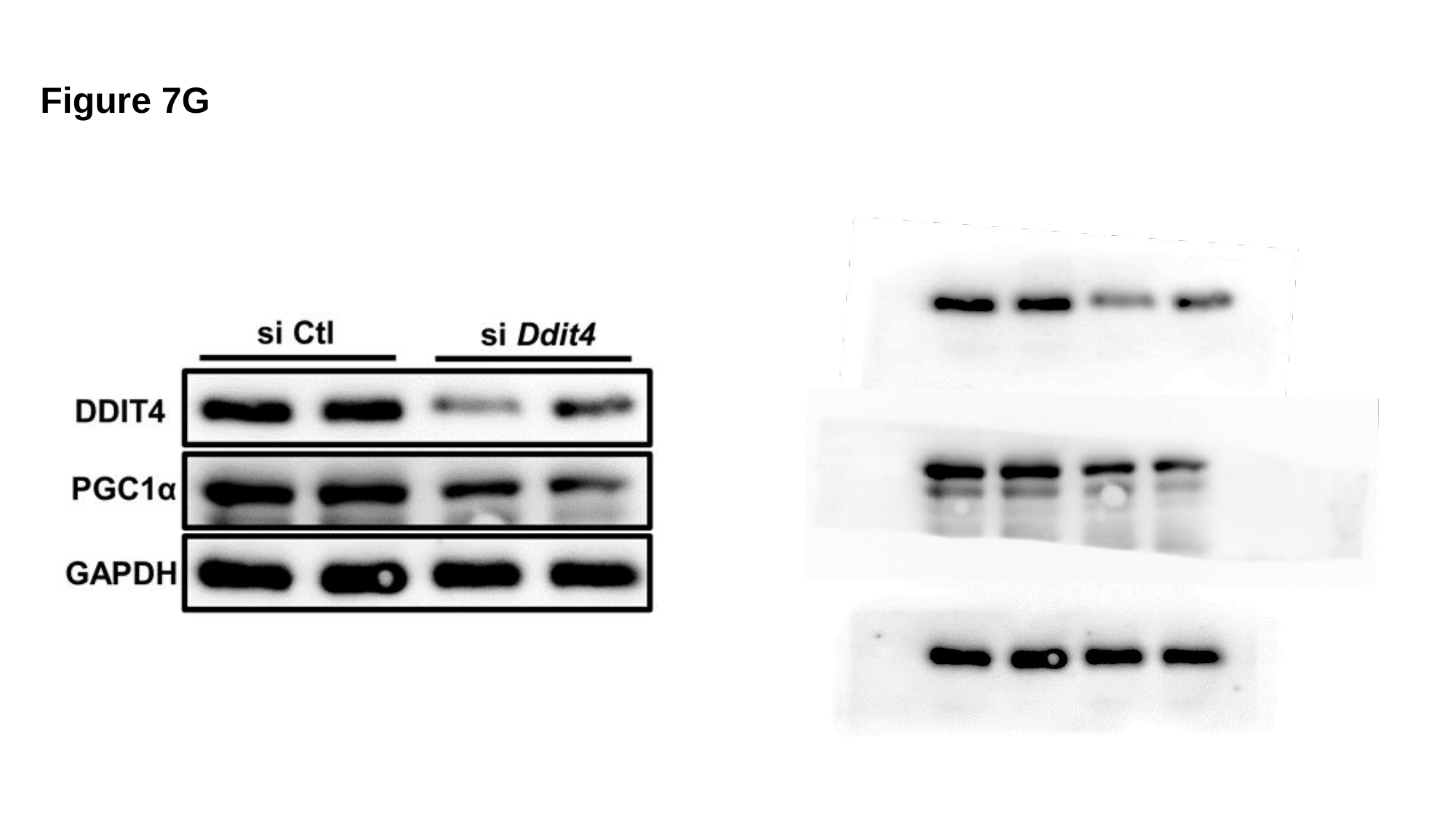

Figure 7G

## Slide 10
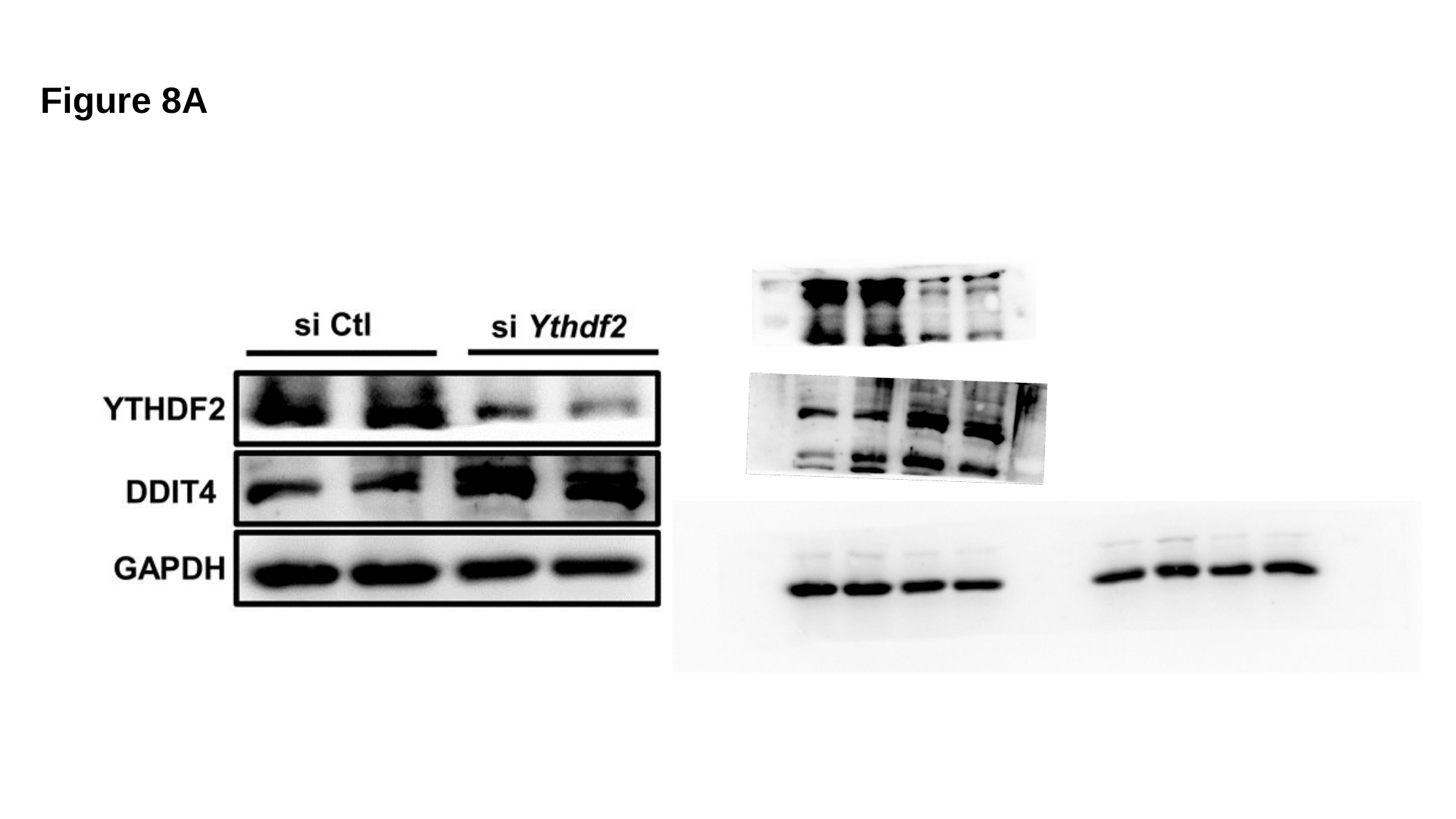

Figure 8A

## Slide 11
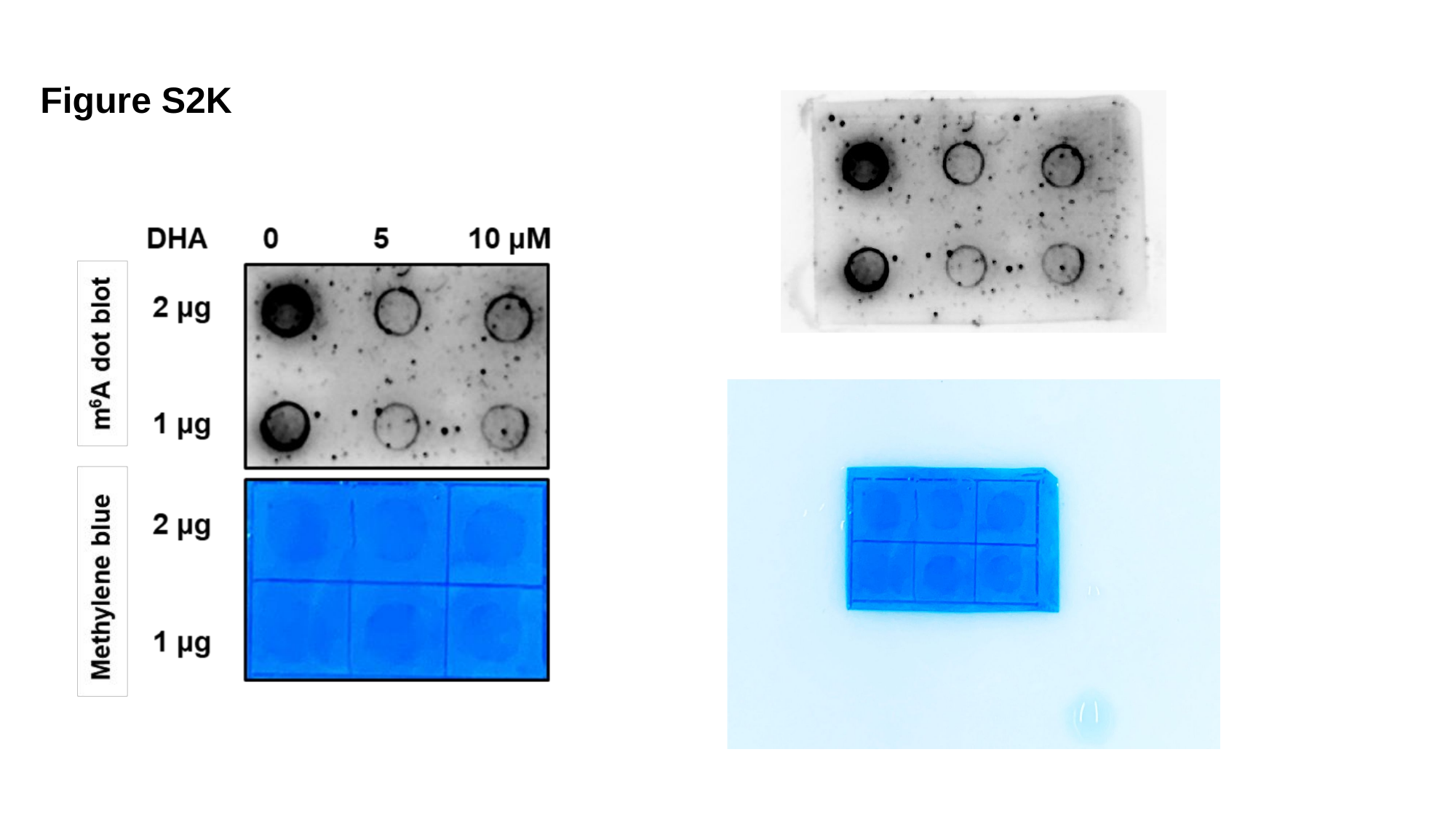

Figure S2K

## Slide 12
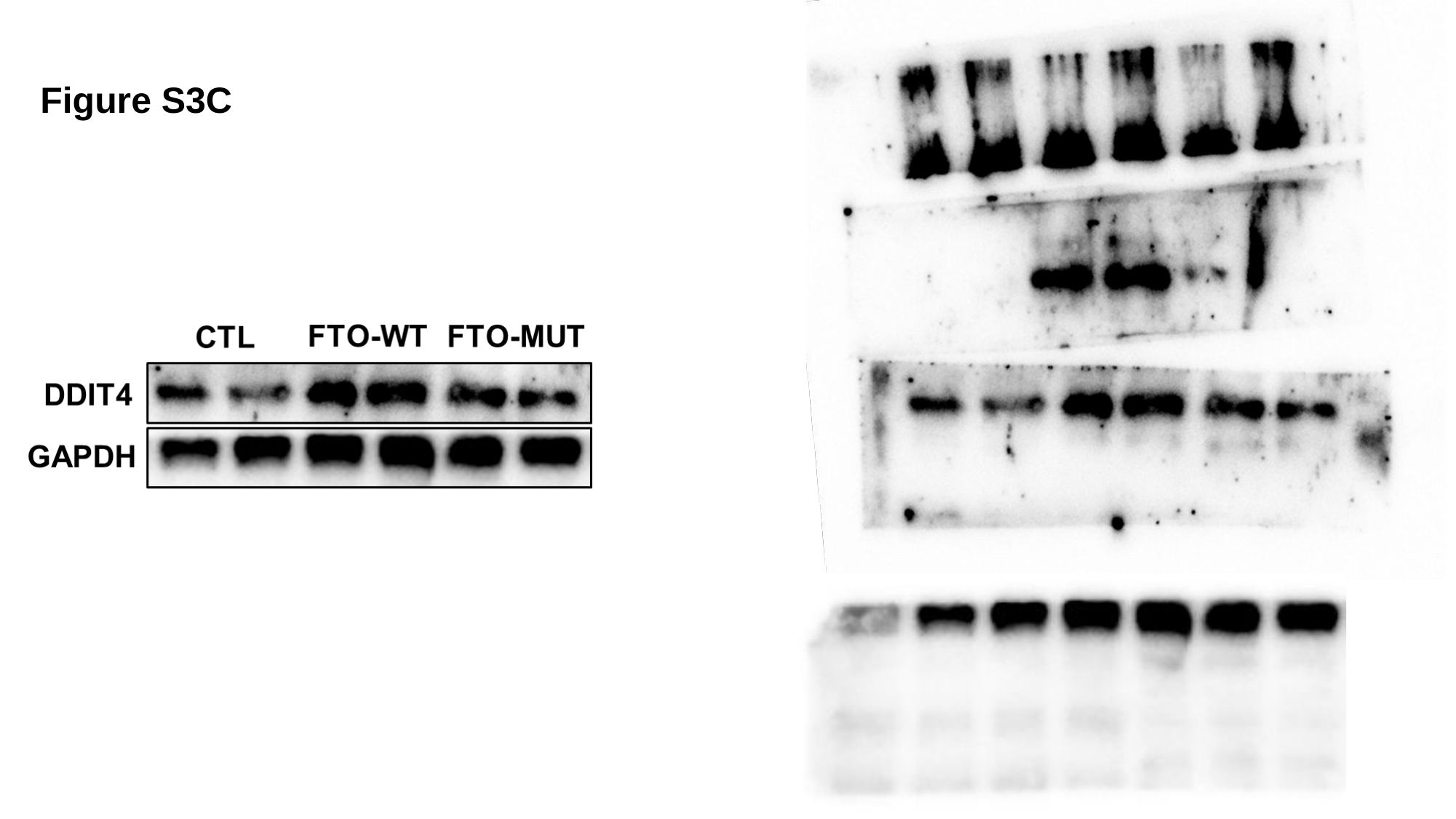

Figure S3C
